# RNA-TVcurve: a Web server for RNA secondary structure comparison based on a multi-scale similarity of its triple vector curve representation

**DOI:** 10.1186/s12859-017-1481-7

**Published:** 2017-01-21

**Authors:** Ying Li, Xiaohu Shi, Yanchun Liang, Juan Xie, Yu Zhang, Qin Ma

**Affiliations:** 1 College of Computer Science and Technology, Jilin University, Changchun, 130012 China; 2 Key Laboratory of Symbolic Computation and Knowledge Engineering (Jilin University), Ministry of Education, Changchun, 130012 China; 3 Zhuhai Laboratory of Key Laboratory of Symbol Computation and Knowledge Engineering of Ministry of Education, Zhuhai College of Jilin University, Zhuhai, 519041 China; 4 Department of Mathematics and Statistics, South Dakota State University, Brookings, SD 57007 USA; 5 Bioinformatics and Mathematical Biosciences Lab, Department of Agronomy, Horticulture and Plant Science, South Dakota State University, Brookings, SD 57007 USA; 6 BioSNTR, Brookings, SD USA

**Keywords:** RNA-TVcurve, RNA structure comparison, Multi-scale similarity, Phylogenetic tree, Numerical representations

## Abstract

**Background:**

RNAs have been found to carry diverse functionalities in nature. Inferring the similarity between two given RNAs is a fundamental step to understand and interpret their functional relationship. The majority of functional RNAs show conserved secondary structures, rather than sequence conservation. Those algorithms relying on sequence-based features usually have limitations in their prediction performance. Hence, integrating RNA structure features is very critical for RNA analysis. Existing algorithms mainly fall into two categories: alignment-based and alignment-free. The alignment-free algorithms of RNA comparison usually have lower time complexity than alignment-based algorithms.

**Results:**

An alignment-free RNA comparison algorithm was proposed, in which novel numerical representations RNA-TVcurve (triple vector curve representation) of RNA sequence and corresponding secondary structure features are provided. Then a multi-scale similarity score of two given RNAs was designed based on wavelet decomposition of their numerical representation. In support of RNA mutation and phylogenetic analysis, a web server (RNA-TVcurve) was designed based on this alignment-free RNA comparison algorithm. It provides three functional modules: 1) visualization of numerical representation of RNA secondary structure; 2) detection of single-point mutation based on secondary structure; and 3) comparison of pairwise and multiple RNA secondary structures. The inputs of the web server require RNA primary sequences, while corresponding secondary structures are optional. For the primary sequences alone, the web server can compute the secondary structures using free energy minimization algorithm in terms of RNAfold tool from Vienna RNA package.

**Conclusion:**

RNA-TVcurve is the first integrated web server, based on an alignment-free method, to deliver a suite of RNA analysis functions, including visualization, mutation analysis and multiple RNAs structure comparison. The comparison results with two popular RNA comparison tools, RNApdist and RNAdistance, showcased that RNA-TVcurve can efficiently capture subtle relationships among RNAs for mutation detection and non-coding RNA classification. All the relevant results were shown in an intuitive graphical manner, and can be freely downloaded from this server. RNA-TVcurve, along with test examples and detailed documents, are available at: http://ml.jlu.edu.cn/tvcurve/.

**Electronic supplementary material:**

The online version of this article (doi:10.1186/s12859-017-1481-7) contains supplementary material, which is available to authorized users.

## Background

RNAs are known to carry important functionalities among diverse species, while those with similar functions are usually less conserved at sequence level in structured regions. Hence, it is critical to consider the structural information in RNA comparison, aiming to elucidate their functional and evolutional relationship. RNA three dimensional structure mainly determine the Current methods for RNA secondary structure comparison can be generally classified into two categories, i.e., alignment-based and alignment-free.

The alignment-based methods are mostly conducted in a dynamic programming framework, relying on a string or tree representation of RNA secondary structure [1–6]. The RNA secondary structure alignments generally fall into two broad categories: Sankoff-based and Non-Sankoff RNA alignment. The Sankoff algorithm simultaneously folds and aligns two or more RNA sequences using free energy minimization [6]. Its time complexity and space complexity are O (n^3M^) and O (n^2M^), respectively, given M RNA sequences with lengths of n. Although this algorithm has a very good performance in prediction, its heavy computational complexity greatly limits its application.

Therefore, in order to be more practical, there are many simplifications of Sankoff algorithm [7] including but not limited to Consan [2], Dynalign [8, 9], PMcomp [10], Stemloc [11], Foldalign [12, 13], locARNA [14], SPARSE [15], MARNA [16], FoldAlignM [17], Murlet [18], CARNA [19] and RAF [20]. PMcomp performs pairwise and progressive multiple alignments based on base pairing probability matrices computed from McCaskill’s algorithm [21], which simplifies Sankoff’s model by predicting only a single consensus structure. LocARNA improves PMcomp using local alignment. CARNA is an improved algorithm to consider RNA pseudoknot structures based on the PMcomp model. SPARSE specifies prediction and alignment of RNAs based on their structure ensembles in quadratic time. Dynalign calculates a common structure by combining free energy minimization and comparative sequence analysis to find a low free energy structure common to two sequences without any sequence identity. Non-Sankoff algorithms separate these two processes: folding and alignments. The main methods include CMfinder [22], LARA [23], RNAdistance [4], RNAStrAt [24], RNAforester [25], SCARNA [26], gardenia [27], ERA [28] Web-Beagle [29] and RNApdist [30]. RNAdistance, RNAforester, RNApdist and RNAStrAt are all tree-based approaches. RNAdistance compares RNA secondary structures based on tree edit distance, and RNAforester calculates the pairwise- or multiple-alignment of structures based on tree alignment. RNApdist is another program based on the base pairing probabilities, which has been included in the Vienna RNA package [31]. RNAStrAt uses a conservative edit distance and mapping between two RNA stem-loops based on a tree representation. LARA is a graph-based representation for structural alignments using an integer linear program. CMfinder is based on a covariance model (CM). Gardenia is based on the definition of the common arc-annotated supersequence. ERA is an efficient and accurate RNA secondary structure alignment tool using the sparse dynamic programming technique. In [32], a new context-aware encoding representation for RNA secondary structure named as BEAR is designed and an RNA structural alignment based on BEAR encoding is proposed by integrating a constructed substitution matrix of RNA structural elements with Needleman-Wunsch algorithm. The web server Web-Beagle [29] of RNA structural alignment based on BEAR encoding is developed. Among the above alignment-based tools, RNAdistance and RNApdist are the two most widely-used tools, which have been included in the Vienna RNA package [31]. It is noteworthy that the above alignment-based methods have an intensive requirement in time complexity.

An alignment-free algorithm is usually based on a numerical representation of sequences, which requires a novel idea and alternative way to visualize, analyze and compare DNAs, RNAs and proteins. Hence, compared to the alignment-based methods for RNA structure comparison, the studies on alignment-free algorithms are limited. In [33], various graphical representations of protein, DNA and the secondary structure of RNA were reviewed. A three-dimensional curve that constitutes a unique representation of DNA sequence was proposed by Chun-ting Zhang [34], so-called Z-curve. It is one of the representative studies in this field and has been successfully applied to many different bioinformatics areas, including identification of replication origin, proteins-coding genes, genomic island, GC content variations, and phylogenetic analysis [35–37]. As far as we known, noncoding RNAs are more conserved at structure levels [38]. It is very important to consider the structure features for alignment-free RNA comparison algorithm. The sequence and structure features of RNAs have been changed into different representations, including 2 dimensions, 3 dimensions, 4 dimensions and image [39–46]. For instance, the features of the sequence and base pairing were transformed into a linear sequence, then the Lempel-Ziv algorithm was applied to compute the similarity [45]. GraphClust is another alignment-free algorithm based on a fast graph kernel technique for clustering of local RNA secondary structures [46]. Recently, signal and image processing methods have been found useful in elucidating the similarity, and more details could be found in [47]. Alignment-free methods for RNA comparison usually have lower time complexity and less accuracy compared with alignment-based methods.

In addition, with steadily increasing numbers of known 3D RNA structures, some 3D structure alignment algorithms and their web serves including SETTER [48, 49], MultiSETTER [50, 51], R3Dalign [52, 53], Rclick [54, 55], R3D-BLAST [56] and R3D-2-MSA [57] have been developed.

We developed a web server, RNA-TVcurve, for inferring RNAs’ relationship based on comparing their secondary-structures. The underlying alignment-free algorithm is summarized in the next section and more details can be found in [58]. This method has two unique features: 1) a novel numerical representation of RNA sequence and its secondary structure; and 2) a novel multi-scale similarity metric of above numerical representation based on wavelet decomposition. RNA-TVcurve has three functions: 1) visualization of RNA secondary structure; 2) detection of single-point mutation based on RNA secondary structure; and 3) construction of phylogenetic trees for multiple RNAs. Evaluations of our in-house algorithm and web server has been carried out on single point mutation identification for RNA virus and phylogenetic trees construction for non-coding RNA families. These performance comparison analyses were only conducted between RNA-TVcurve and two alignment-based programs, RNAdistance and RNApdist, as no other web servers based on alignment-free methods for RNA structure comparison are available in the public domain. The results showcased that RNA-TVcurve is more reliable than RNAdistance and RNApdist. The main results are shown using intuitive figures. All the related results and execution files can be freely downloaded at http://ml.jlu.edu.cn/tvcurve/.

## Implementation

### Methodology for pairwise RNA secondary structure comparison based on RNA-TVcurve

**Fig. 1:**
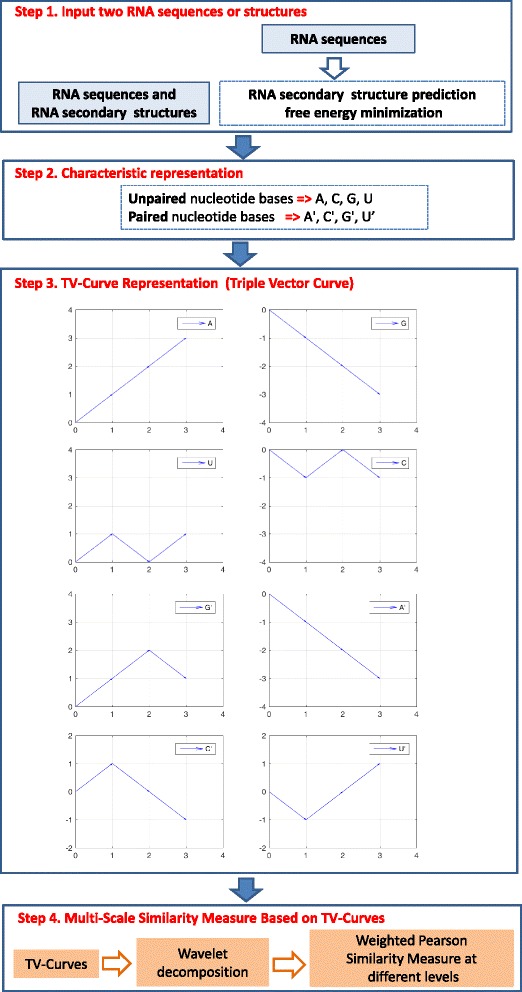
Core methodology of RNA-TVcurve including four sub-block diagram: (1) RNA secondary structure prediction, (2) Characteristic representation for combining RNA sequence and secondary structure, (3) Construction of TV-Curve (Triple Vector Representation), (4) Wavelet decomposition of TV-Curves and multi-scale similarity measure based on TV-Curves

The details of the underlying algorithm of RNA-TVcurve can be found in [58]. Here we firstly summarize the core methodology for pairwise RNA secondary structure comparison in the following four steps (Fig. 1).

**Step 1**: The minimum free energy method in terms of Vienna’s RNAfold [59] is used to predict the secondary structure for any given RNA sequence if users do not provide secondary structure.

**Step 2**: The characteristic representation of RNA secondary structure is designed based on eight symbols, in which A, C, G and U were used for the unpaired nucleotide bases, and A′, C′, G′, and U′ were used for the same bases if paired by hydrogen bonds.

**Step 3**: A new numerical representation of RNA is constructed, named as Triple vector curves (TV-curve), by integrating RNA sequence and secondary structure features. The triple vectors are designed to represent the eight A, T, C, G, A′, U′, G′ and C′ symbols respectively as follows:

$$ \begin{array}{l}\left(1,1\right),\left(1,1\right),\left(1,1\right)\Rightarrow \mathrm{A},\left(1,-1\right),\left(1,-1\right),\left(1,1\right)\Rightarrow {\mathrm{A}}^{\hbox{'}}\\ {}\left(1,1\right),\left(1,-1\right),\left(1,1\right)\Rightarrow \mathrm{U},\left(1,-1\right),\left(1,1\right),\left(1,1\right)\Rightarrow {\mathrm{U}}^{\hbox{'}}\\ {}\left(1,1\right),\left(1,-1\right),\left(1,-1\right)\Rightarrow \mathrm{G},\left(1,1\right),\left(1,1\right),\left(1,-1\right)\Rightarrow {\mathrm{G}}^{\hbox{'}}\\ {}\left(1,-1\right),\left(1,1\right),\left(1,-1\right)\Rightarrow \mathrm{C},\left(1,1\right),\left(1,-1\right),\left(1,-1\right)\Rightarrow {\mathrm{C}}^{\hbox{'}}\end{array} $$

The schematic diagram of triple vectors representing eight letters is shown in subfigure of Step 3 in Fig. 1. There is a one-to-one mapping between the TV-curves and the RNA information of sequences and secondary structures. The TV-curve is a visualization method to represent the information of the primary and secondary structure of an RNA molecular, which can provide users another angle to display and infer the difference between RNAs, especially for the long RNAs.

**Step 4**: Firstly, the wavelet decomposition [60] with *L*-level of RNA TV-Curves are computed. After the *L*-level transformation, the detailed coefficients and approximation coefficients at the different resolution are obtained. *L* is usually taken as four by default. Next, a novel similarity metric, named multi-scale similarity, was designed to capture both the global and local properties of the constructed TV-curve based on wavelet decomposition. The Pearson correlation coefficients at different decomposition levels are calculated and the weighted sum is taken in the multi-scale similarity calculation.

### Detection of maximal structure difference among all RNA single point mutants

For an RNA sequence of length *N*, there are three possible types of mutations for each position. For the original RNA sequence, if the original structure is not provided, the secondary structure of the original RNA sequence will be predicted with the minimum free energy algorithm. All the 3*N single point mutated RNA sequences are constructed. For each single point mutated RNA sequence, the minimum free energy algorithm is used to predict the secondary structure. In order to identify the maximal structural mutation, the similarity between each mutated RNA sequence and original RNA sequence based RNA-TVcurve is computed. The similarity score is changed into a distance score by one minus it. The mutation with the maximal structural distance is identified. Furthermore, the histogram of the structure distance scores of all the single point mutation is provided to help users to comprehensively understand the landscape of all the single point mutation. In addition, to describe the structural stability of each position, the deleterious profile is defined as the maximum distance in structures between the wild-type and the possible single point mutants at each position. The structurally important sites can be easily identified by the peaks with a larger structural distance on the profile. The distributions of the distances between the wild-type and all the possible single mutants are shown as histograms. The histograms of the distances between the wild-type and all the possible single mutants provide the visual understanding on RNA mutational robustness.

### Construction of RNA phylogenetic trees

The main steps of construction the RNA phylogenetic tree are listed as follows:

**Step 1**: Compute TV-curve for each sequence in a given RNA set.

**Step 2**: Calculate similarity matrix of N (N-1)/2 similarity between all pairs of N sequences by pairwise RNA secondary structure comparison based on RNA-TVcurve as mentioned above.

**Step 3**: Converting raw similarity scores to distances scores by 1-minus-similarity.

**Step 4**: Construct a phylogenetic tree from the distance matrix by UPGMA (Unweighted Pair Group Method with Arithmetic Mean). Firstly, cluster a pair of RNAs (taxa) with the smallest distance. Then re-calculate an average distance between the new cluster and other taxa, obtaining a new distance matrix. Finally, repeat previous step until clusters converge.

### Usage of Web server

RNA-TVcurve offers four different functional modules (Additional file 1: Figure S1): (1) ***TVCurve***: visualization and generation of TV-curve for a provided RNA sequence; (2) ***RNA Mutation***: RNA single point mutation detection for a given RNA sequence; (3) ***RNA Multiple***:construction of RNA phylogenetic tree for multiple RNAs. (4) ***RNA Pairwise***: *perform pairwise comparison based on RNA-TVcurve.* All these usages are freely available and no login information is required.

The server is accessible at: http://ml.jlu.edu.cn/tvcurve/ with standard web browsers (Mozilla Firefox, Google Chrome, Internet Explorer, Safari). Each functional module of the web serve of RNA-TVcurve has been tested through four examples. The test samples for each module and the examples shown in this paper are listed in the server and also included in Additional file 2, aiming to help users to understand and use each of the functional modules. The input of the web server can be either entered in a text box or uploaded as an individual file from a local computer. Users can either directly run example datasets on the web server or upload their files to test different functional modules. The minimal length of the input RNA sequence for RNA-TVcurve is 10.

All functional modules require the input in FASTA format or a modified FASTA including the secondary structure in dot-bracket. Upon a job is submitted to the server, a job ID will be generated. A user can just save the job ID and use it to extract the results. For a submitted job, its results are saved up to 1 month so that the user can query and retrieve. The probable long-running jobs include the RNA structural mutation detection for a longer RNA sequence with a length larger than 1000 nt, and the RNA phylogenetic tree construction for more than 300 RNA sequences. Some important results including the TV-curve, the mutated structure with the maximal structural difference and the phylogenetic tree are shown in a more intuitive graphical manner. The output page also offers the download of the results as a single packed file in “.zip” format for offline analysis. The README file in the packed file provides information on of the downloaded results. The detailed steps and functionality of each module are introduced in the following.

#### Functional module 1:TVCurve

This module provides a visualization for the TV curve constructed from provided RNA sequences and secondary structures. The input should be RNA sequences in the FASTA format that are provided with or without secondary structure information. If the structures of some RNAs are not provided, the secondary structures are predicted with the minimum free energy algorithm. The output provides the TV-curve representation and the multi-scale decomposition of the TV-curve, and the secondary structure for each RNA. All the results including TV-curve representation, the multi-scale decomposition of the TV-curve, and the secondary structure can be freely downloaded. Detailed executive process and results of the example for this module are presented in Additional file 3: Figure S2. The example dataset uses nine virus RNA sequences.

#### Functional module 2:RNA mutation

This module is to accomplish the RNA single point structural mutation detection. The required input for this module is a single RNA sequence and structure or a sequence alone. If multiple RNA sequences are submitted, only the first sequence is calculated. For this functional module, there is an option “Compare with RNA distance and RNApdist” to benchmark the results with both tools. This option is not selected by default. The maximum distance in structures between the wild-type and those with mutations at each position is extracted into a structural deleteriousness profile. This module provides the deleteriousness profiles and their histograms for RNA-TVcurve. The structure with the maximum structure difference calculated by RNA-TVcurve is given the corresponding mutation types and mutation sites are also provided. In addition, in order to help users to visualize the structural difference between the wild-type and all the mutants, the secondary structures of the wild-type RNA and the mutated RNA with the maximum structural distance are also displayed on the web page. All the structural distance scores of the single point mutations detected by RNA-TVcurve are ranked, which are included in the downloaded file. If users select the option “Compare with RNA distance and RNApdist”, this module provides the deleteriousness profiles and their histograms for RNA-TVcurve, RNAdistance and RNApdist, respectively. The structure with the maximum structure difference calculated by RNA-TVcurve, RNAdistance and RNApdist are given and the corresponding mutation types and mutation sites are also provided. We will rank all the structural differences of the single point mutations detected by the three methods respectively. The detailed executive process and results of the example for this module are presented in Additional file 4: Figure S3. The input example dataset is the Leptomonas collosoma sequence.

#### Functional module 3: RNA multiple

For Module 3, the input is a set of RNA sequences and their structures or RNA sequences and part of structures or primary sequences alone (at least three RNA sequences are required). The phylogenetic tree computed by RNA-TVcurve is displayed on the web page. All the related results include the phylogenetic tree, TV curves and distance matrix using RNA-TVcurve can be freely downloaded. Similar to the module of “RNA Mutation”, users have an option “Compare with RNA distance and RNApdist”. If this option is selected, the three phylogenetic trees computed by RNA-TVcurve, RNApdist and RNAdistance are all shown in an intuitive graphical manner on the web page, which can help users make comparison and further analysis. In this case, the downloaded file includes three phylogenetic trees and distance matrix computed by RNA-TVcurve, RNApdist and RNAdistance. When the number of the sequences is larger, the alignment-based RNApdist needs more time to compute. In addition, the labels of the leaves on the tree are hard to read when the number of multiple RNAs is large. We construct the tree at horizontal direction in order to more clearly display the labels of the leaves on the tree when the number of multiple RNAs is larger than 20.

The example of construction the phylogenetic trees for multiple RNAs is 100 sequences of four non-coding RNA families including miRNA, RNaseP_arch, 5S_rRNA and tRNA. In order to make the performance for each method clearly, four types of colors are marked for each family. The phylogenetic tree by RNA-TVcurve has four noticeable branches and only four RNAs are assigned at the wrong branch, where each branch represents one RNA non-coding family. The detailed executive process and results of the example for this module are presented in Additional file 5: Figure S4.

#### Functional module 4: RNA pairwise

This module is to accomplish RNA pairwise comparison based on RNA-TVcurve. The input of this module includes two sets of RNA sequences. One set is named as query set and the other one as target sets. Both of the two sequence sets include RNA sequences and their structures or RNA sequences and part of structures or primary sequences alone. They should be in the FASTA format and contains the same number of RNAs. In this module, the RNAs in the query set and the corresponding RNAs in the target set are compared by RNA-TVcurve. The distance score for each pair of RNAs are shown on the web server. In addition, the RNA TV curve and secondary structure of each sequence are also displayed on the web server to help users further understand the structure difference between a pair of RNAs. All the related results are packed in a zip file for download.

The detailed executive process and results of the example for this module is presented in Additional file 6: Figure S5. The pairwise comparison is conducted between query set and target set with 3 virus sequences respectively.

## Results

We assessed the performance of RNA-TVcurve compared with the two popular alignment-based programs, RNAdistance and RNApdist. In order to systematically evaluate the performance of RNA-TVcurve, two types of experiments are designed, one of which is to test the capability to infer the evolution for different species and the other is to validate the performance to distinguish the different types of RNA families.

### Type I study: 5S rRNAs, RNase P and RNase MRP

**Fig. 2:**
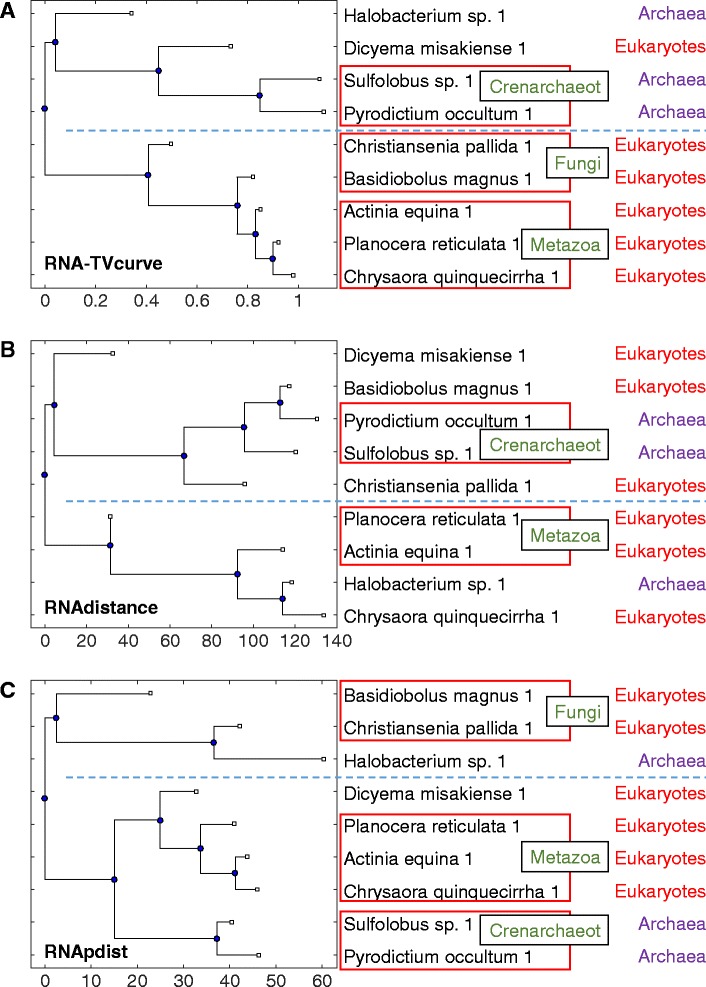
The phylogenetic tree for the set I of 5S rRNAs. **a** the phylogenetic tree constructed by RNA-TVcurve, **b** the phylogenetic tree constructed by RNAdistance, **c** the phylogenetic tree constructed by RNApdist. In this figure RNAs in same the box mean they are consistent to the same known subgroup. The line is to mark the branch of the constructed tree

**Fig. 3:**
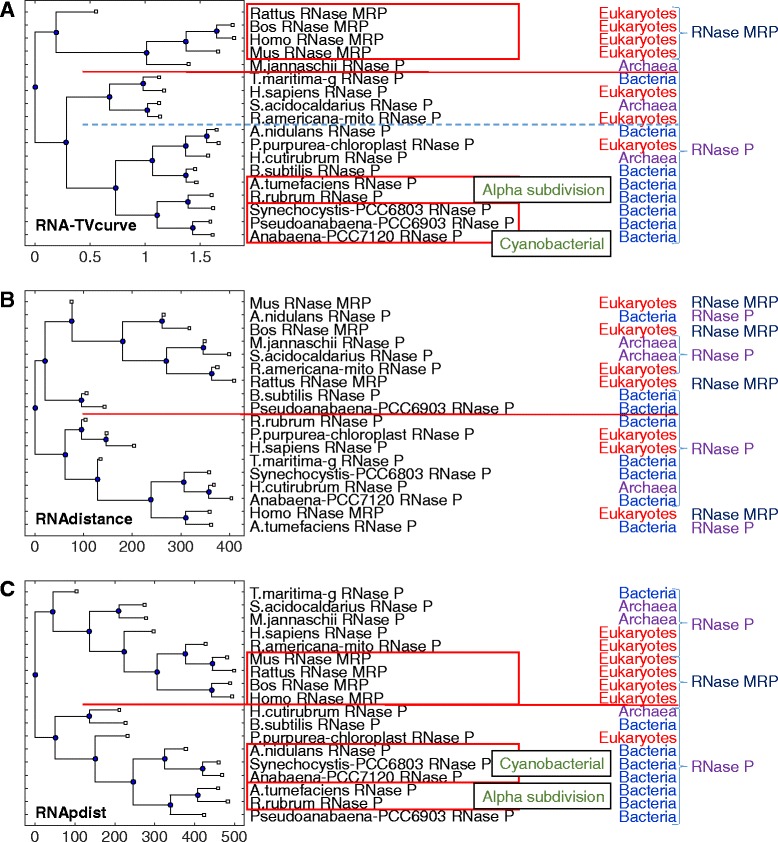
The phylogenetic tree for the set II of RNase P and RNase MRP. **a** the phylogenetic tree constructed by RNA-TVcurve, **b** the phylogenetic tree constructed by RNAdistance, **c** the phylogenetic tree constructed by RNApdist. In this figure RNAs in same the box mean they are consistent to the same known subgroup. The line is to mark the branch of the constructed tree

The sets of different types of RNAs from different species in [45] are designed to evaluate the performance of the constructed RNA phylogenetic tree in terms of its accuracy. In order to clearly identify the evolutional relationship among the above sets, Additional file 7: Table S1 and Table S2 provided the detailed information of family and species for the following two sets: set I from 5S Ribosomal RNA Database [61]; and set II including RNase P and RNase MRP obtained from the RNase P Database [62] and NCBI. Set II is used to test distantly related sequences, which have limited homology information. The phylogenetic tree for set I of 5S rRNAs constructed by RNA-TVcurve, RNAdistance and RNApdist is shown in Fig. 2. The phylogenetic trees constructed by RNA-TVcurve has clearly two branches for the groups of *Archaea* and *Eukaryotes*, only Dicyema misakiense is wrongly placed on the Archaea branch, however the other two phylogenetic trees constructed by RNAdistance and RNApdist do not show such good property. In addition, on the phylogenetic trees constructed by RNA-TVcurve and RNApdist, the three groups of Fungi, Metazoa and Crenarchaeot are all placed closely. The phylogenetic trees for set II of RNase P and RNase MRP constructed by the three programs are listed in Fig. 3. RNA-TVcurve also shows the best result compared with RNApdist and RNAdistance, in which the phylogenetic tree constructed by RNA-TVcurve has clearly two branches for the groups of RNase MRP and RNase P, only one RNase P *M.jannaschii* is wrongly placed on the branch of RNase MRP. The groups of Alpha subdivision and Cyanobacterial are all grouped closely. The other two phylogenetic trees constructed by RNAdistance and RNApdist have limited power in distinguishing the RNase MRP and RNase P.

### Type II study: different RNA families from rfam database

**Fig. 4:**
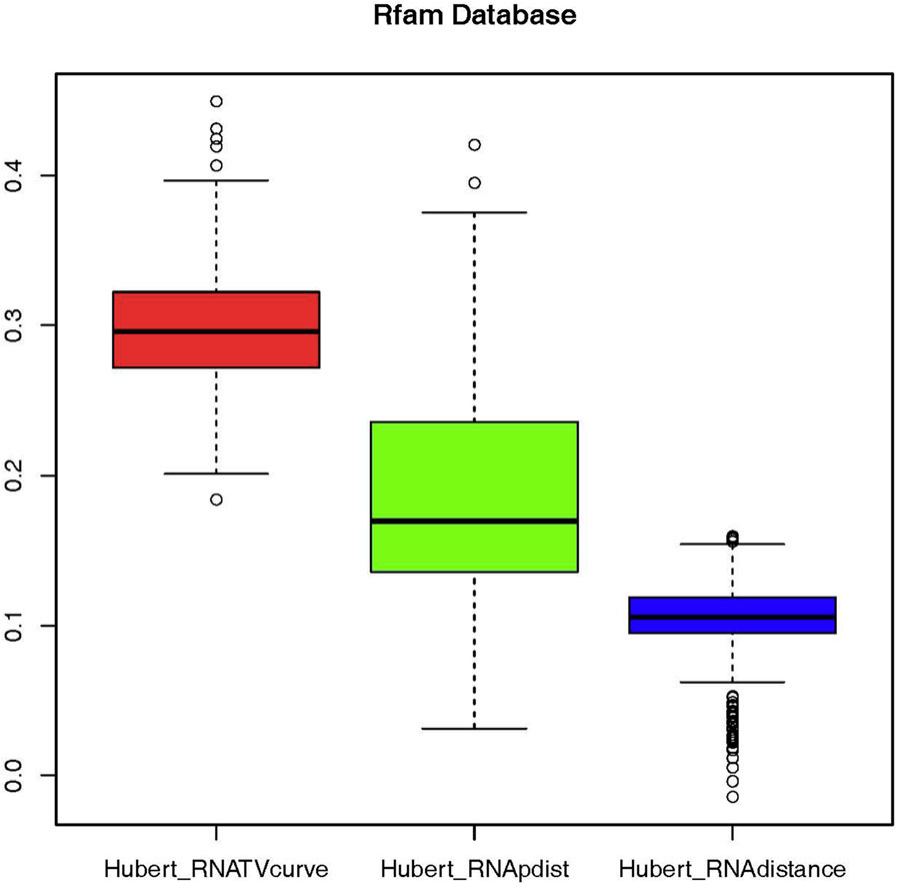
The comparison of Pearson correlation coefficients for RNA-TVCurve, RNApdist and RNAdistance to validate the ability to distinguish the different RNA families from Rfam Database

Next, different RNA families from Rfam database are used to evaluate the ability of these three programs in distinguishing the different types of RNA families. The sequences of rRNA, tRNA, miRNA and RNase_P families are downloaded from the Rfam database. In order to directly compare the performance for distinguishing the different types of RNAs, Pearson correlation is used to measure the consistency between the similarity measure and the background co-membership matrix. It is actually the correlation between the similarity matrix computed and the background co-membership matrix [58]. Intuitively, a larger value of Pearson correlation means a higher consistency with the background matrix. Due to the large amount of sequences including 19,111 RNA sequences, it is not realistic to compute the similarities among them all. In this paper, we made 5,000 random samplings from the downloaded sequences, in which 100 sequences from each RNA family are randomly selected. Then the similarity matrices for all the families were computed by RNA-TVcurve, RNAdistance and RNApdist. The corresponding Pearson correlation coefficients were calculated for each sampling. The time complexity of RNApdist and RNAdistance are both O (*n* ^3^). RNA-TVcurve dramatically reduce the computational time complexity to O (*n*) [63], where *n* is the length of an RNA sequence. The comparison of Pearson correlation coefficients of RNA-TVcurve, RNAdistance and RNApdist for the 5,000 experiments is shown in Fig. 4. RNA-TVcurve is showcased significantly superior to RNApdist and RNAdistance.

## Conclusions

The RNA-TVcurve server provides an access to our alignment-free RNA secondary structure comparison algorithms. It is the first integrated web server for RNA analysis based on alignment-free method, which provides a novel visualization of RNA secondary structure, mutation analysis, pairwise and multiple RNAs secondary structure comparison. This method can provide a novel view to visualize and analyze RNAs. Our evaluations suggest that the web server is a good visualization tool, and more importantly, it can be used to solve a wide range of RNA related analysis and mining problems. Particularly, it is very useful for making deleterious mutation prediction, RNA structural feature extraction and multiple RNA structural comparison. We believe the server will be a useful tool for RNA comparison and fill a void in this field.

A future improvement could be RNA-TVcurve capacity extension to handle multiple point mutations. In addition, the native secondary structure of an RNA is often a suboptimal structure not the predicted structure with minimum free energy due to limitations of thermodynamic models. Integrating multiple predicted suboptimal structures in the RNA structural similarity measurement is still very challenging and there is still a big room for improvement in its performance. In further study, we will focus on developing an RNA comparison tool based on integration of multiple suboptimal structural features. Since real structures of RNAs are very close to the structures with the minimum free energy, it is very import to consider a reasonable filter process. In addition, we will develop a new tool and a web server to search the public RNA datasets such as Human 3′ UTR, Mouse 3′ UTR, Human lncRNAs, Mouse lncRNAs, and Structured Rfam based on RNA-TVcurve. These computational techniques will be very useful and crucial for in-depth inference of RNA functions.

## Additional files

Additional file 1: Figure S1. The web server of RNA-TVcurve including four functional modules. A) Main Home page, B) TVCurve, C) RNA Mutations, D) RNA Multiple, and E) RNA Pairwise. (PDF 361 kb)
Additional file 2: Supplementary_example_set: the fasta files of the used RNA sequences. (ZIP 8 kb)
Additional file 3: Figure S2. Schematic diagram of functional Module TVCurve: A) Select the example sequence and the operation button, B) Waiting interface: Job ID, C) Results of the module of TVCurve: results and download interface. (PDF 763 kb)
Additional file 4: Figure S3. Schematic diagram of functional Module RNA Mutation: A) Select the example sequence and the operation button, B) Waiting interface: Job ID, C) Results of the module of RNA Mutation: results and download interface. (PDF 645 kb)
Additional file 5: Figure S4. Schematic diagram of functional Module RNA Multiple: A) Select the example sequence and the operation button, B) Waiting interface: Job ID, C) Results of the module of RNA Multiple: results and download interface. (PDF 934 kb)
Additional file 6: Figure S5. Schematic diagram of functional Module RNA Pairwise: A) Select the example sequence and the operation button, B) Results of the module of RNA Pairwise: results and download interface. (PDF 438 kb)
Additional file 7: Table S1. The information of families and species of Set 1 of 5S rRNA. **Table S2.** The information of families and species of Set 2 of RNase P and RNase MRP. (DOC 49 kb)

## Abbreviation

RNA-TVcurve: RNA triple vector curve representation
