# Supplementary material for: RNA-TVcurve: a Web server for RNA secondary structure comparison based on a multi-scale similarity of its triple vector curve representation
Source: BMC Bioinformatics. 2017 Jan 21;18:51. doi: 10.1186/s12859-017-1481-7 (PMC5251234; doi:10.1186/s12859-017-1481-7)

A. Main Home page

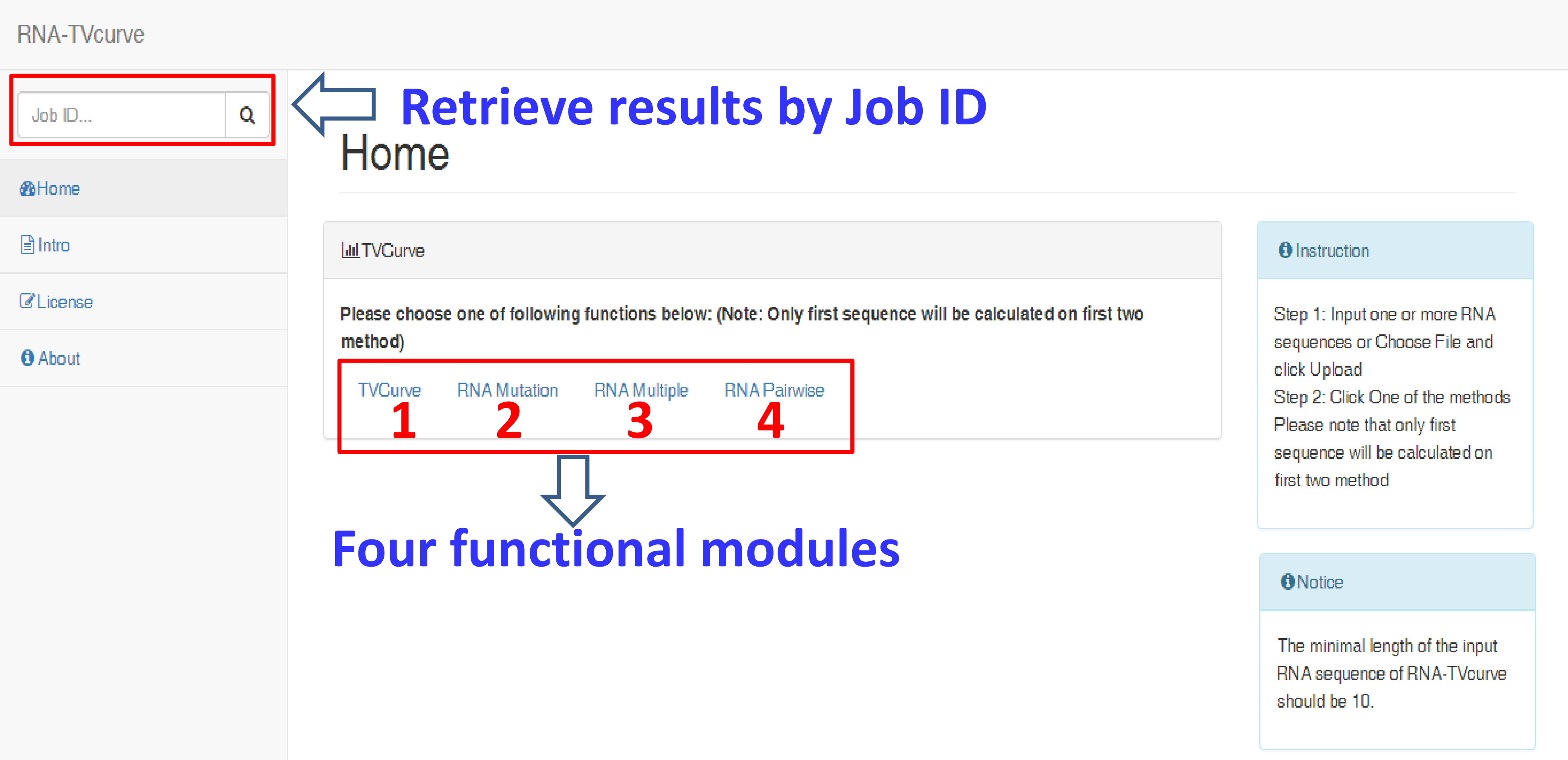

B. Functional module 1: TVCurve

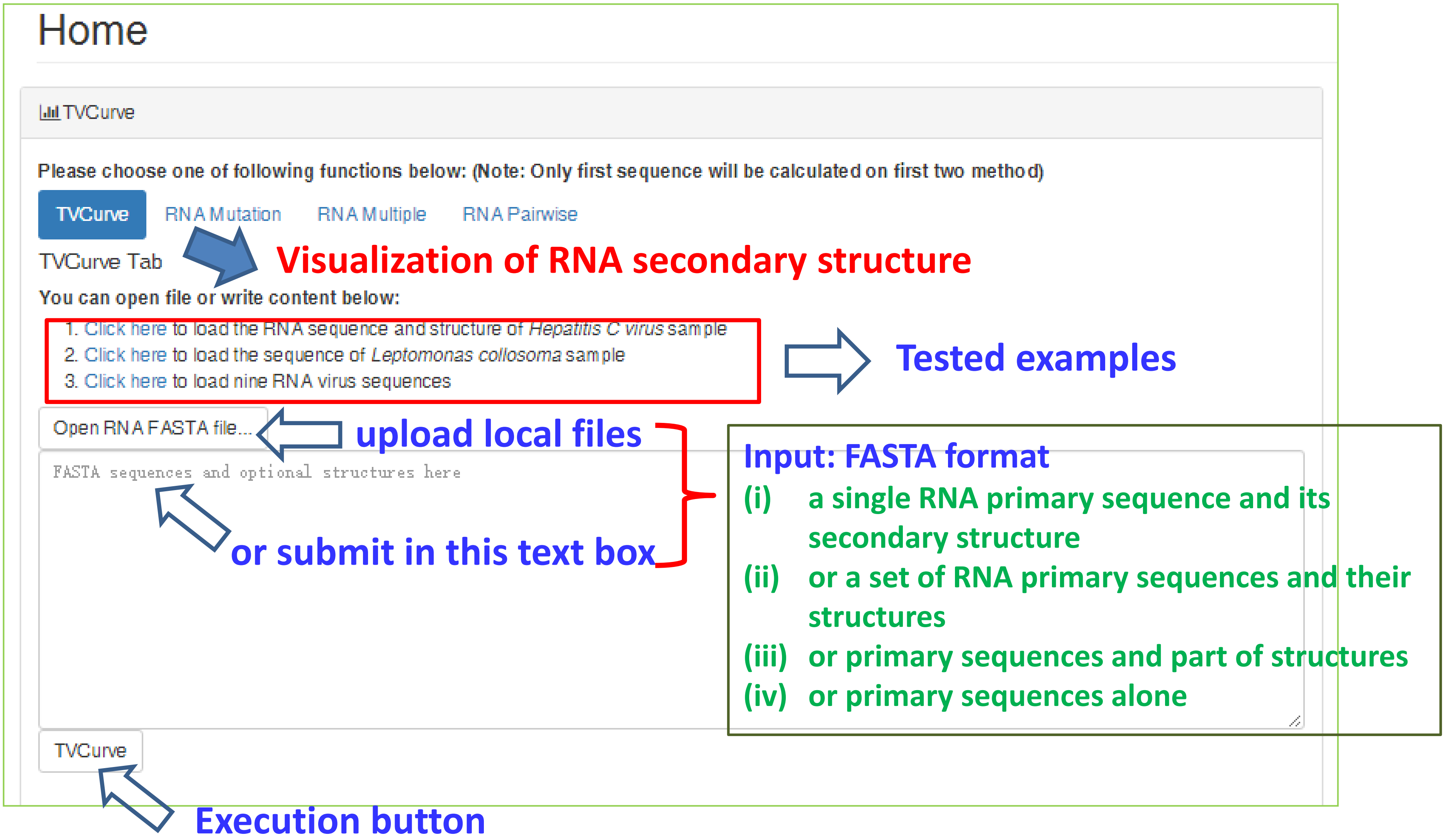

C. *Functional module 2:* RNA Mutation

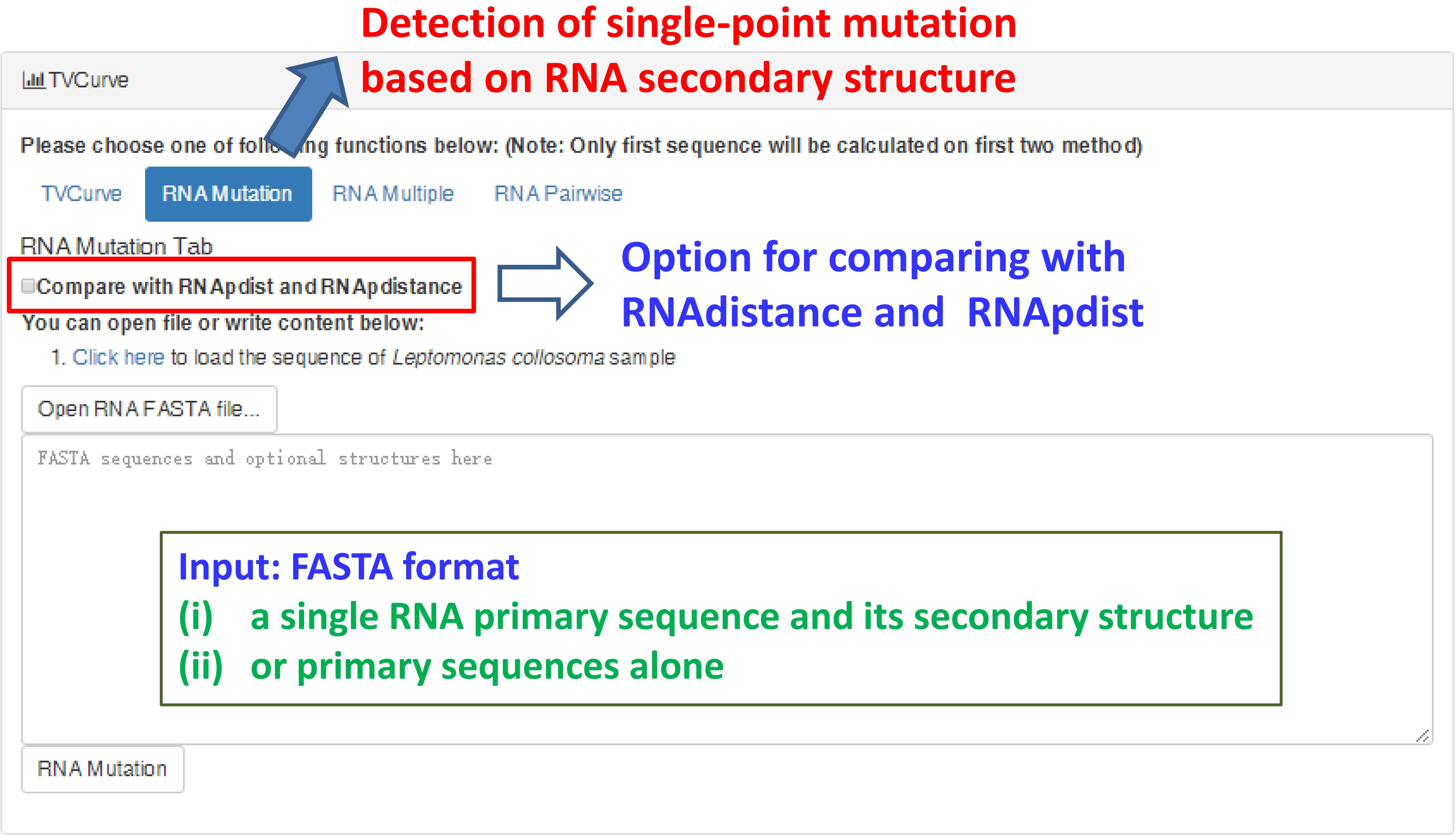

D. Functional module 3: RNA Multiple

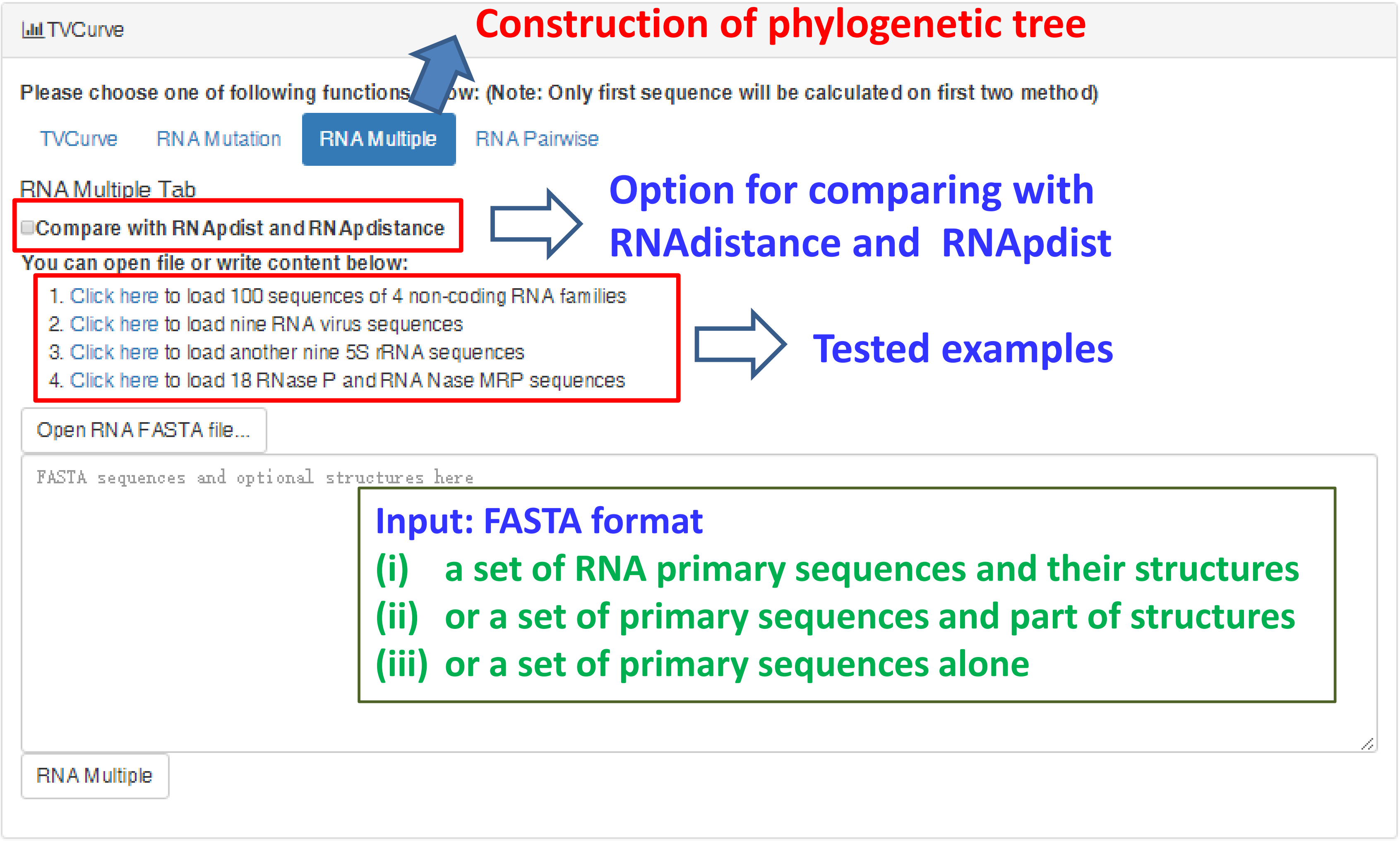

E. Functional module 4: RNA Pairwise

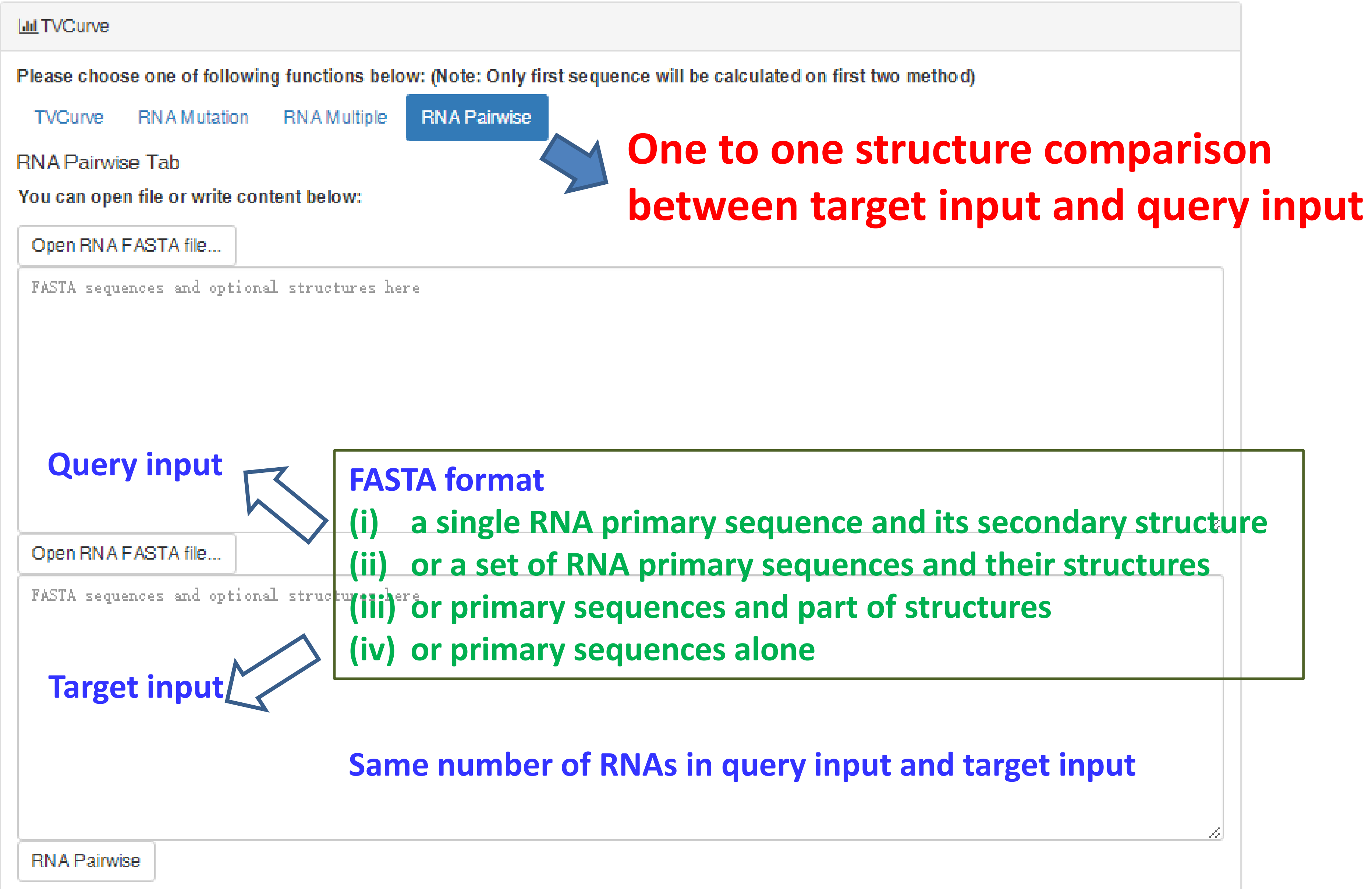

Supplement: Additional file 1: Figure S1. — The web server of RNA-TVcurve including four functional modules. A) Main Home page, B) TVCurve, C) RNA Mutations, D) RNA Multiple, and E) RNA Pairwise. (PDF 361 kb) [file 12859_2017_1481_MOESM1_ESM.pdf]
