# Supplementary material for: RNA-TVcurve: a Web server for RNA secondary structure comparison based on a multi-scale similarity of its triple vector curve representation
Source: BMC Bioinformatics. 2017 Jan 21;18:51. doi: 10.1186/s12859-017-1481-7 (PMC5251234; doi:10.1186/s12859-017-1481-7)
Supplement: Additional file 3: Figure S2. — Schematic diagram of functional Module TVCurve: A) Select the example sequence and the operation button, B) Waiting interface: Job ID, C) Results of the module of TVCurve: results and download interface. (PDF 763 kb) [file 12859_2017_1481_MOESM3_ESM.pdf]

A. Select the example sequence and the operation button

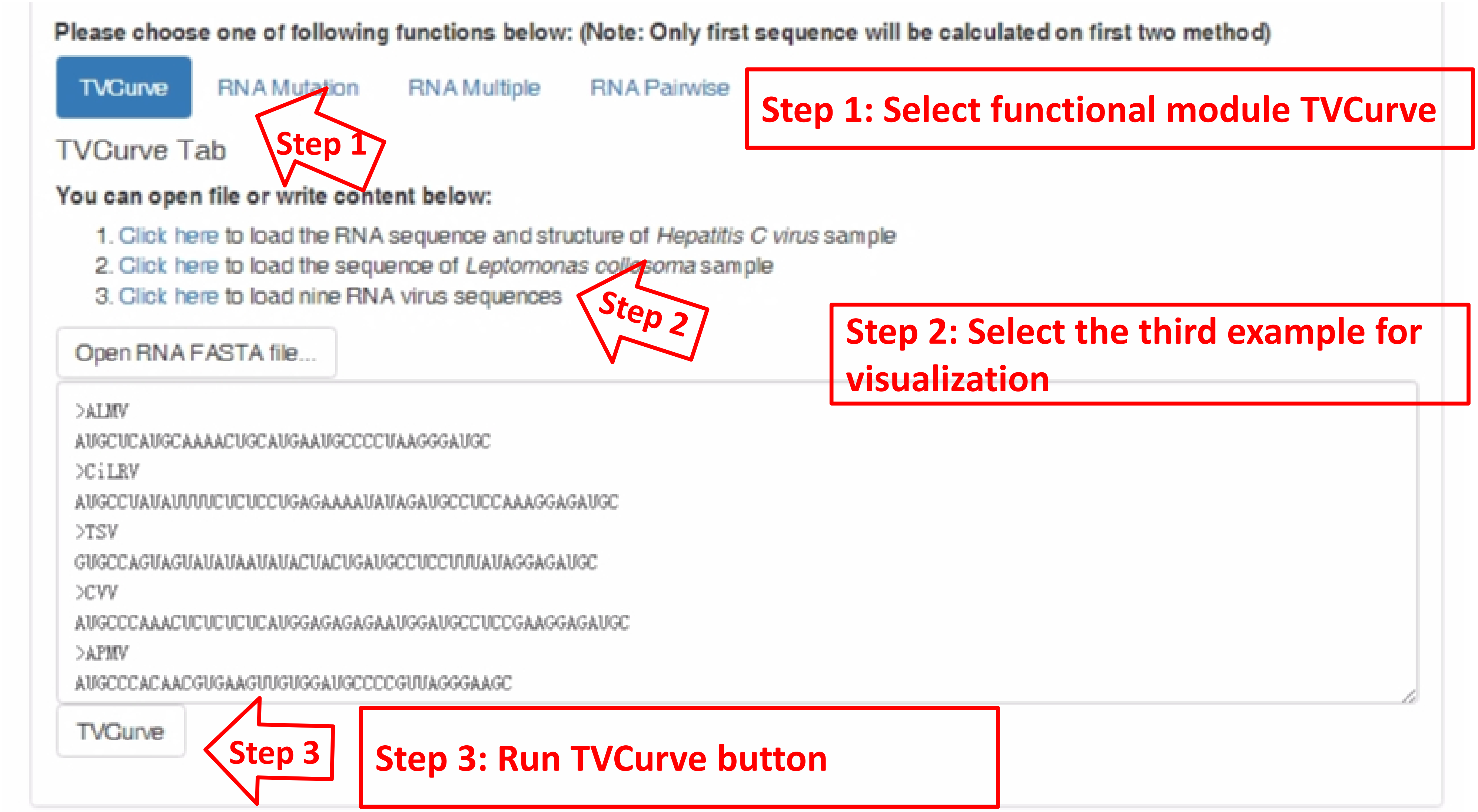

B. Waiting interface: Job ID

Job ID: tv_616c4ba72cee1e4f347f31f42ed4bfa42560ca60

Job ID

Sequecne and Structure

```
>ALMV
AUGCUCAUGCAAAACUGCAUGAAUGCCCCUAAGGGAUGC
....(((((((....)))))))...(((....)))....
[illegible]
```

Results

Please wait for a while. Alternatively, you can save the address of this page or the JobID to retrieve the results.

C. Results of the module of TVCurve: results and download interface

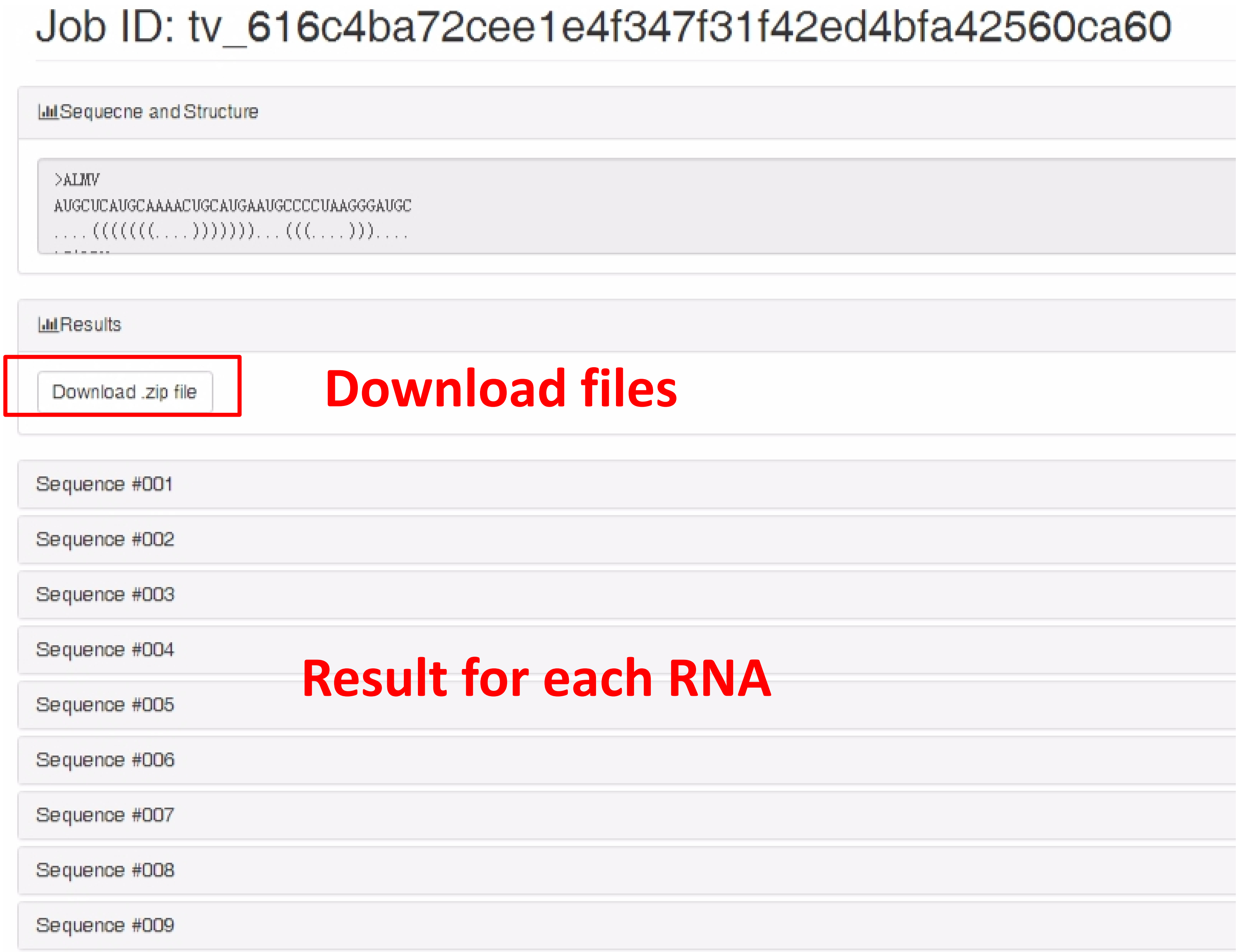

Sequence #001

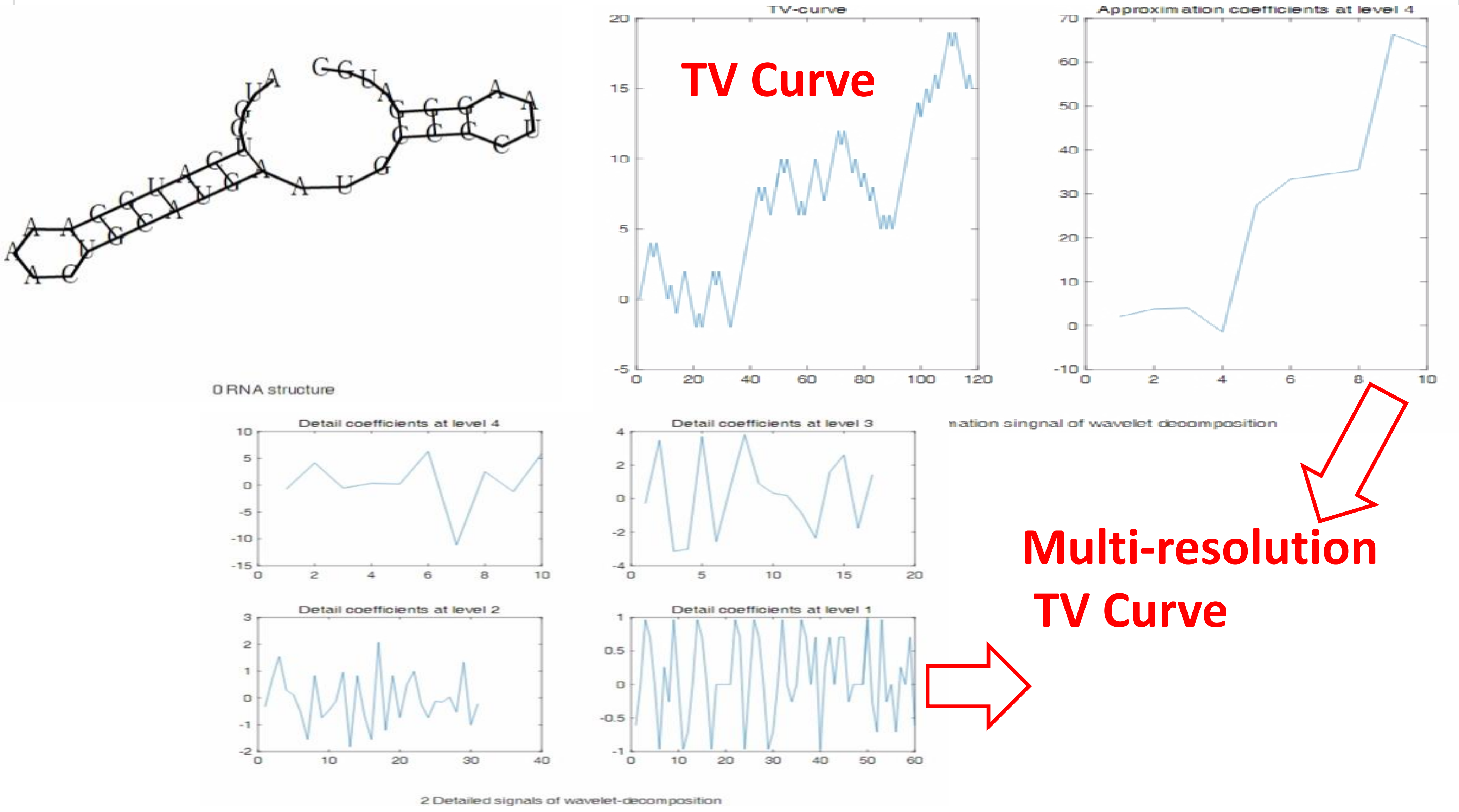


```
> ALMV
AUGCUCAUGCAAAACUGCAUGAAUGCCCCUAAGGGAUGC
....(((((((....)))))))...(((....)))....
```
