# Supplementary material for: RNA-TVcurve: a Web server for RNA secondary structure comparison based on a multi-scale similarity of its triple vector curve representation
Source: BMC Bioinformatics. 2017 Jan 21;18:51. doi: 10.1186/s12859-017-1481-7 (PMC5251234; doi:10.1186/s12859-017-1481-7)
Supplement: Additional file 4: Figure S3. — Schematic diagram of functional Module RNA Mutation: A) Select the example sequence and the operation button, B) Waiting interface: Job ID, C) Results of the module of RNA Mutation: results and download interface. (PDF 645 kb) [file 12859_2017_1481_MOESM4_ESM.pdf]

A. Select the example sequence and the operation button

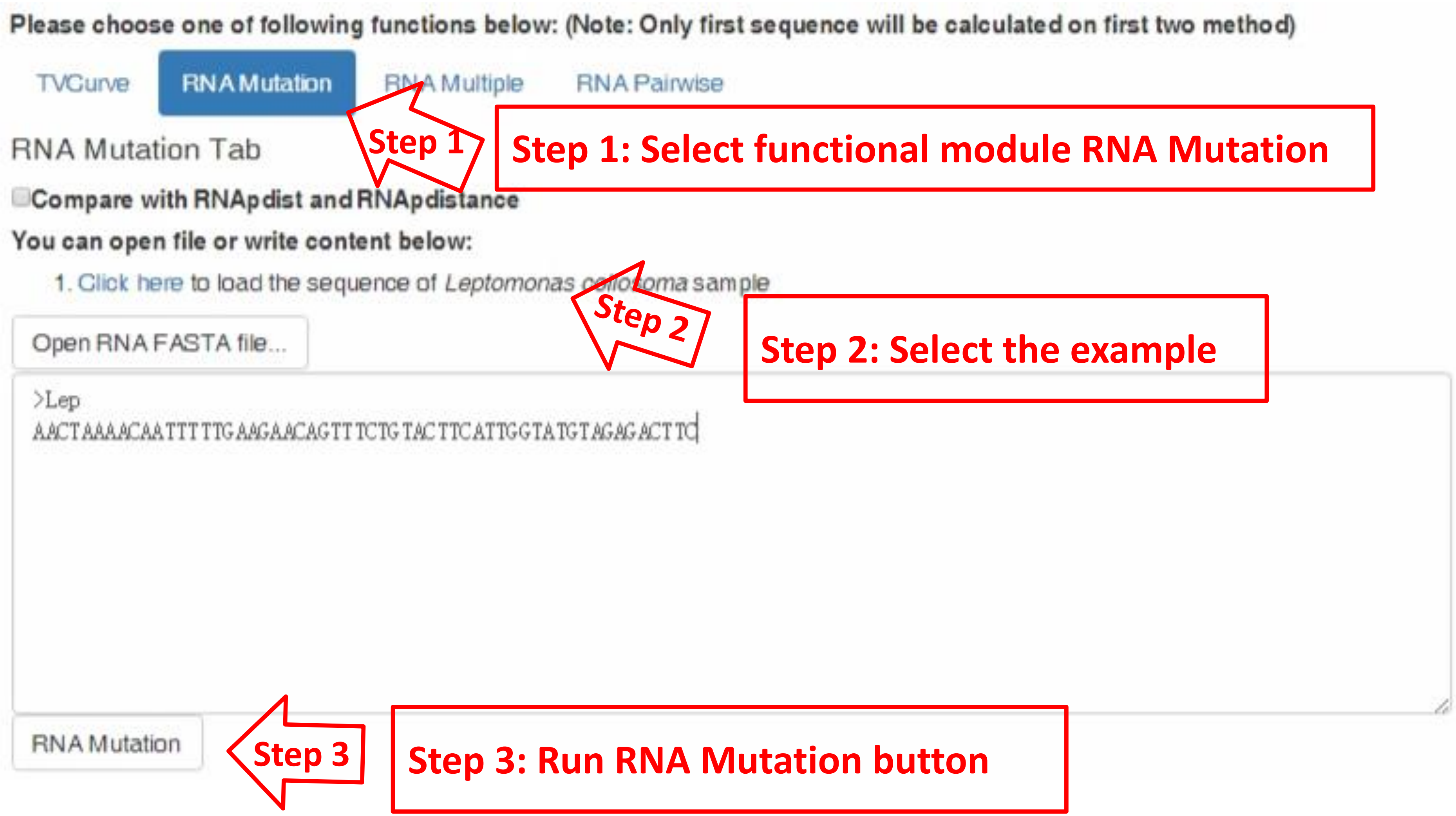

B. Waiting interface: Job ID

Job ID:
mutation_8a720914e7754029009688c2e5296c2f71a14fbf9

Job ID

Sequecne and Structure

```
>Lep
AACUAAAACAAUUUUUGAAGAACAGUUUCUGUACUUCAUUGGUAUGUAGAGACUUC
..((...((((((..(((((.((((...)))).)))))..))).)))..)).....
```

Results

Please wait for a while. Alternatively, you can save the address of this page or the JobID to retrieve the results.

C. Results of the module of RNA Mutation: results and download interface

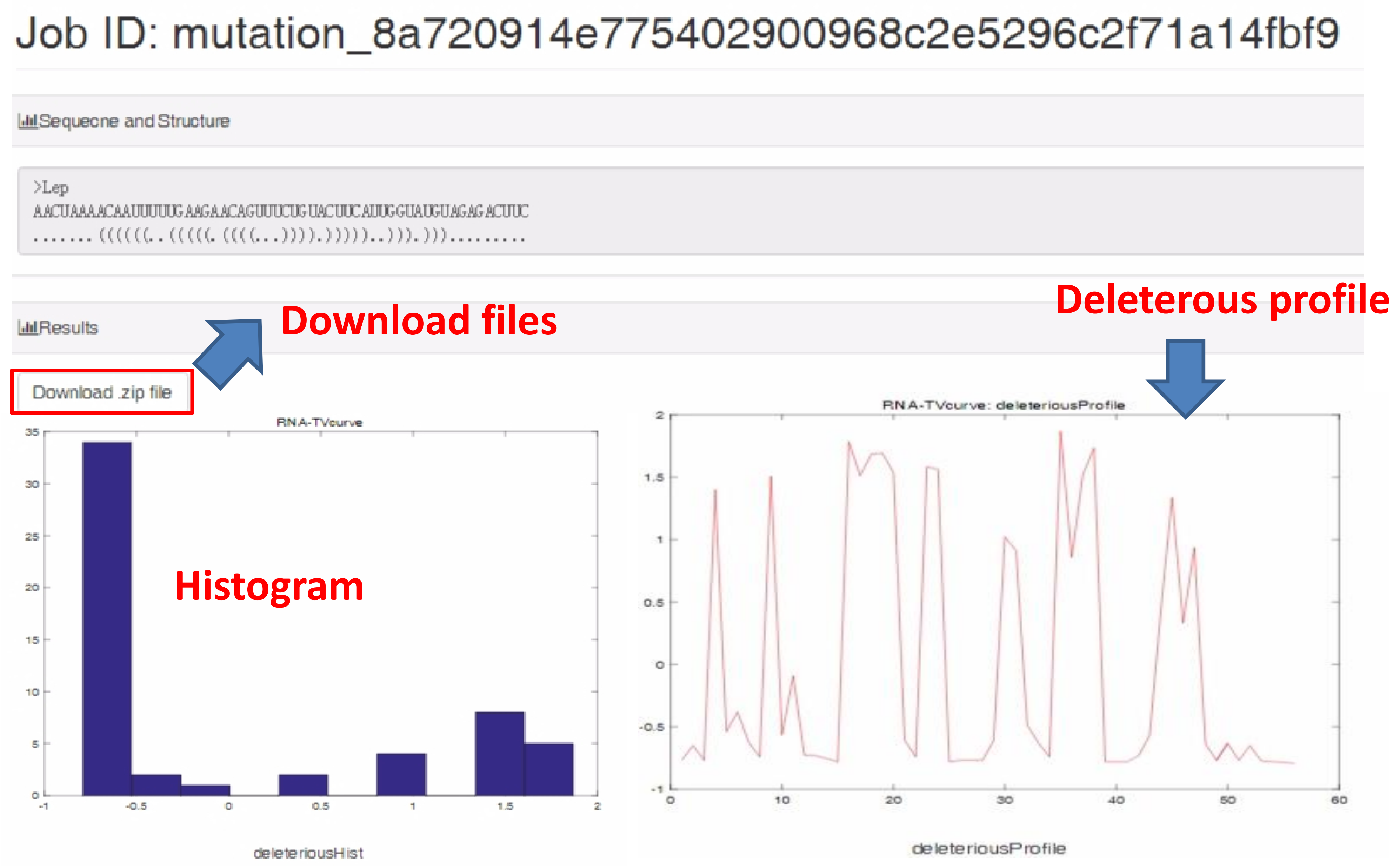

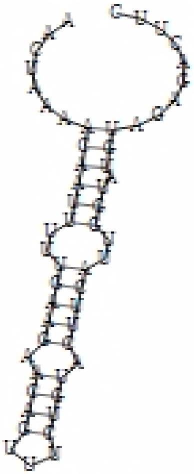

Original

Original RNA structure

```
AACUAAAACAAUUUUUGAAGAACAGUUUCUGUACUUCAUUGGUAUGUAGAGACUUC
.......((((((..(((((.((((...)))).)))))..))).))).........  ( -9.40)
```

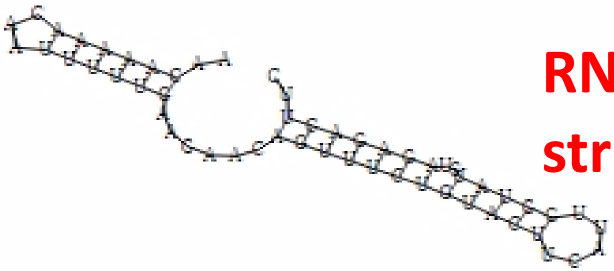

RNA-TVcurve_U4A

RNA U4A mutant with the maximum structure distance

```
AACAAAAACAAUUUUUGAAGAACAGUUUCUGUACUUCAUUGGUAUGUAGAGACUUC
..((((((...))))))......((((((((((((.....)))))..)))))))..  (-10.10)
```
