# Supplementary material for: RNA-TVcurve: a Web server for RNA secondary structure comparison based on a multi-scale similarity of its triple vector curve representation
Source: BMC Bioinformatics. 2017 Jan 21;18:51. doi: 10.1186/s12859-017-1481-7 (PMC5251234; doi:10.1186/s12859-017-1481-7)
Supplement: Additional file 5: Figure S4. — Schematic diagram of functional Module RNA Multiple: A) Select the example sequence and the operation button, B) Waiting interface: Job ID, C) Results of the module of RNA Multiple: results and download interface. (PDF 934 kb) [file 12859_2017_1481_MOESM5_ESM.pdf]

A. Select the example sequence and the operation button

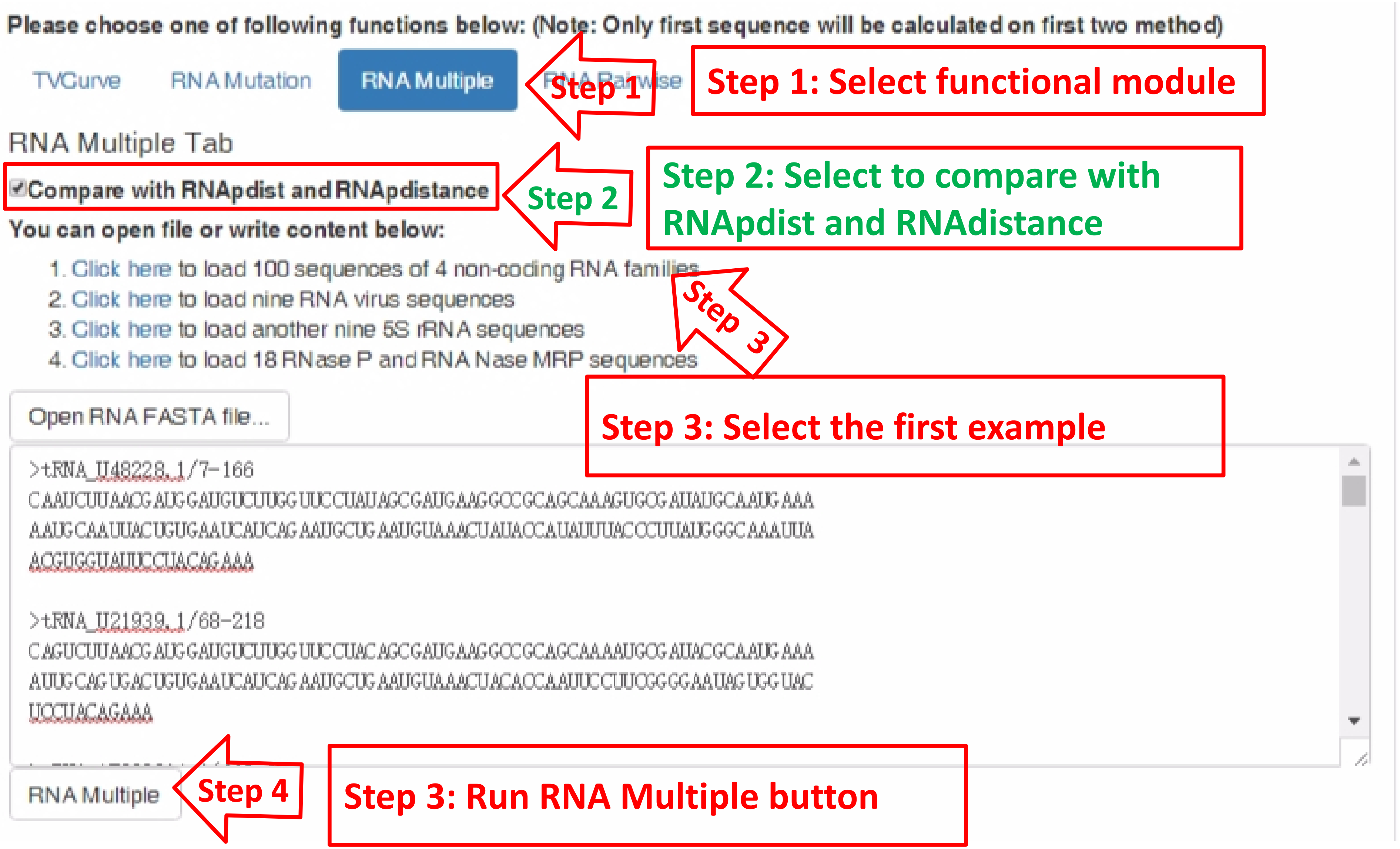

B. Waiting interface: Job ID

Job ID: multiplep_d0fd5247a070b054bd10574b2bb844473b4256ca

Job ID

Sequecne and Structure

>tRNA_U48228.1/7-166
CAAUCUUAACGAUGGAUGUCUUGGUUCCUAUAGCGAUGAAGGCCGCAGCAAAGUGCGAUAUGCAAUGAAAAAUGCAAUUACUGUGAAUCAUCAGAAUGCUGAAUGUAAACUAUACCAUAUUUACCCUUAUGGGCAAAUUAACGUGGUAUUCCUACAGA
AA

Results

Please wait for a while. Alternatively, you can save the address of this page or the JobID to retrieve the results.

C. Results of the module of RNA Multiple: results and download interface

Job ID: multiplep_d0fd5247a070b054bd10574b2bb844473b4256ca

Sequecne and Structure

>tRNA_U48228.1/7-166
CAAUCUUAACGAUGGAUGUCUUGGUUCCUAUAGCGAUGAAGGCCGCAGCAAAGUGCGAUAUGCAAUGAAAAAUGCAAUUACUGUGAAUCAUCAGAAUGCUGAAUGUAAACUAUACCAUAUUUACCCUUAUGGGCAAAUUAACGUGGUAUUCCUACAGA
AA

Results

Download .zip file

Download files

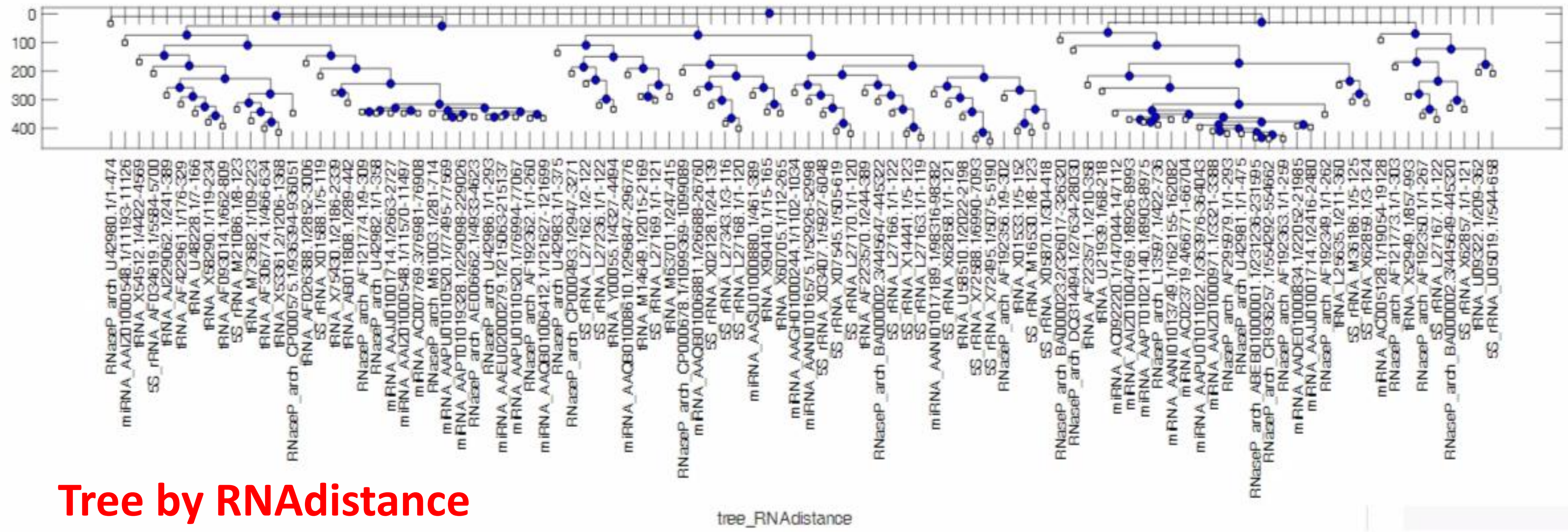

Tree by RNAdistance

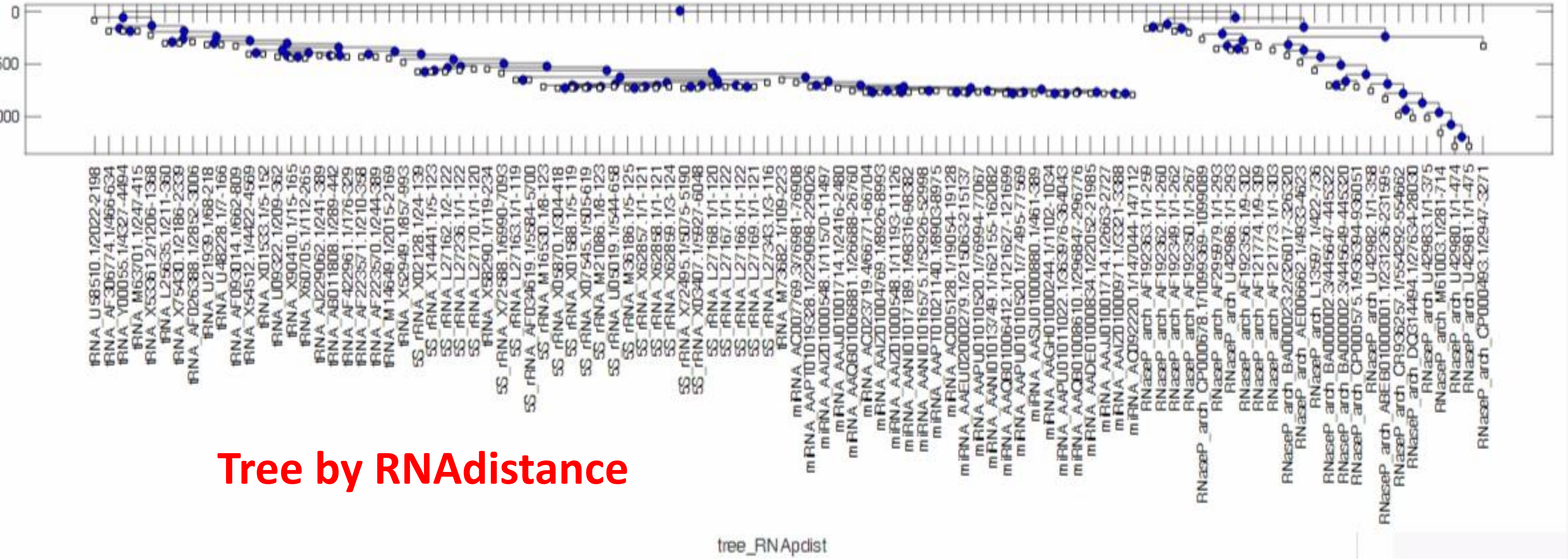
Tree by RNAdistance
tree_RNApdist

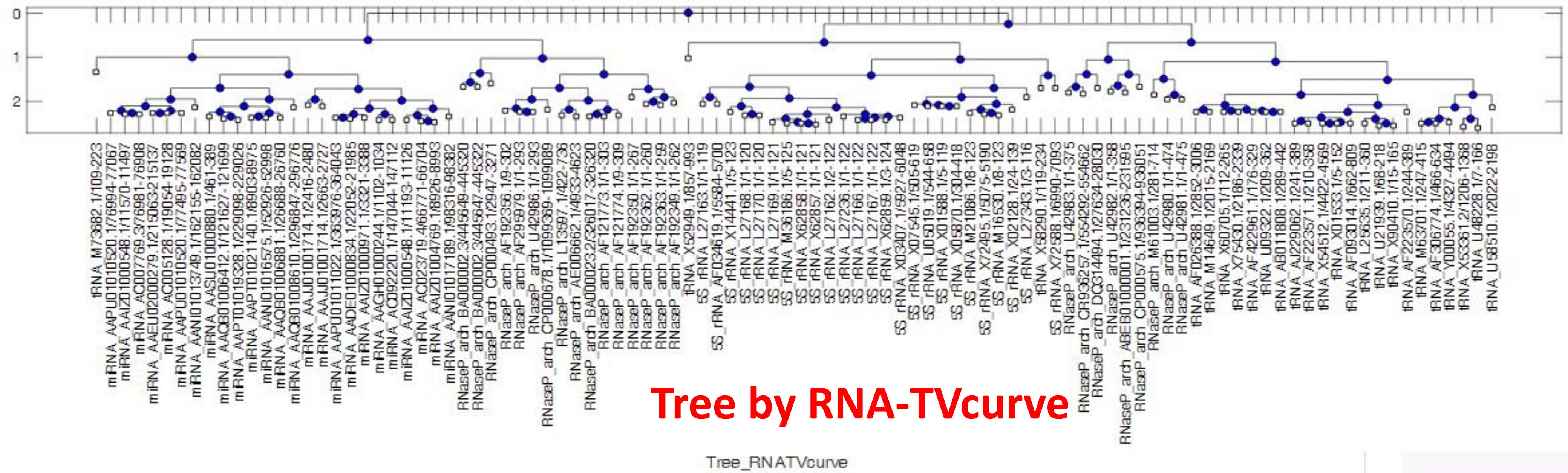
Tree by RNA-TVcurve
Tree_RNATVcurve
