# Supplementary material for: RNA-TVcurve: a Web server for RNA secondary structure comparison based on a multi-scale similarity of its triple vector curve representation
Source: BMC Bioinformatics. 2017 Jan 21;18:51. doi: 10.1186/s12859-017-1481-7 (PMC5251234; doi:10.1186/s12859-017-1481-7)
Supplement: Additional file 6: Figure S5. — Schematic diagram of functional Module RNA Pairwise: A) Select the example sequence and the operation button, B) Results of the module of RNA Pairwise: results and download interface. (PDF 438 kb) [file 12859_2017_1481_MOESM6_ESM.pdf]

A. Select query and target input and the operation button

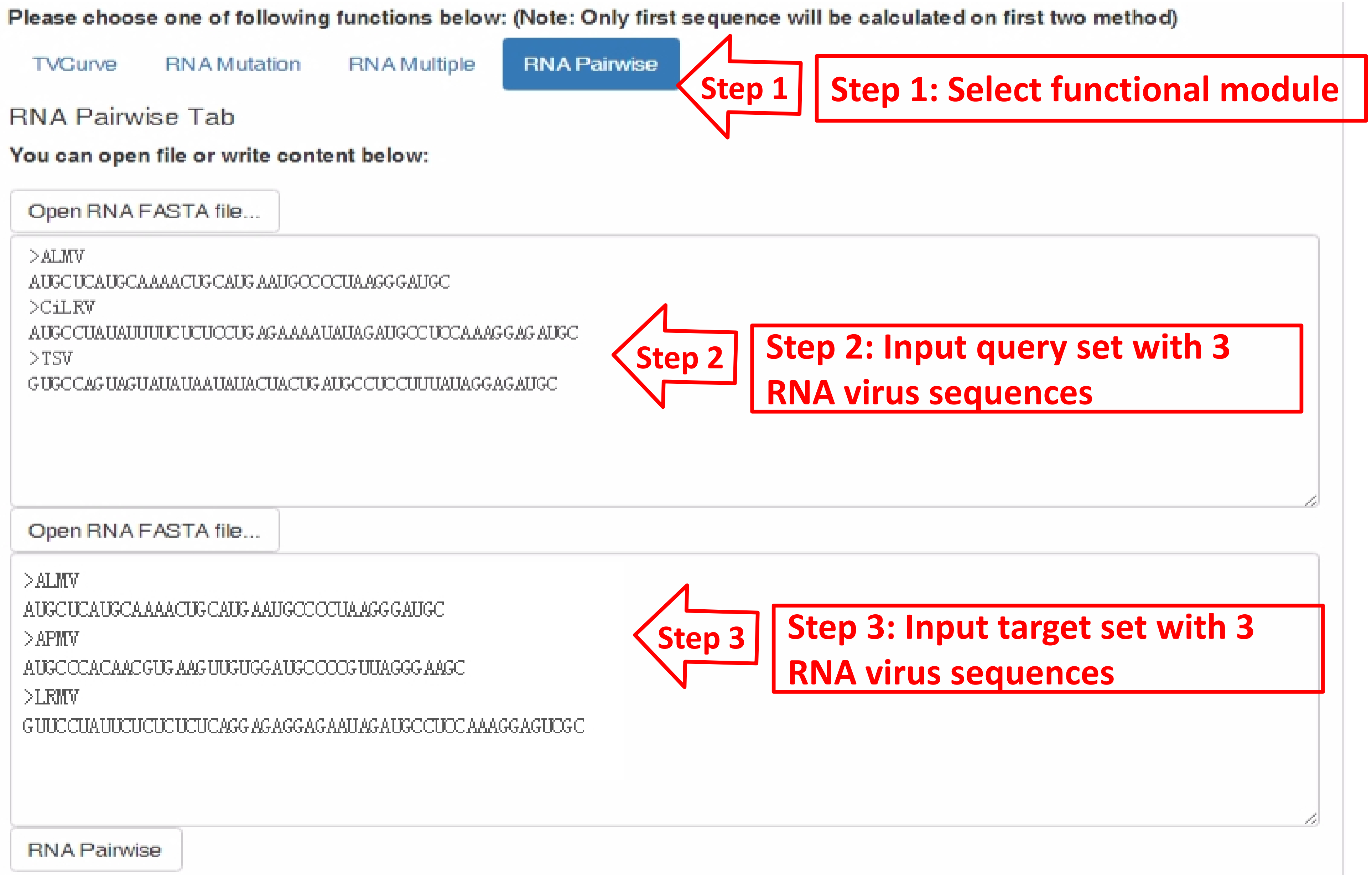

B. Results of the module of RNA Pairwise: results and download interface

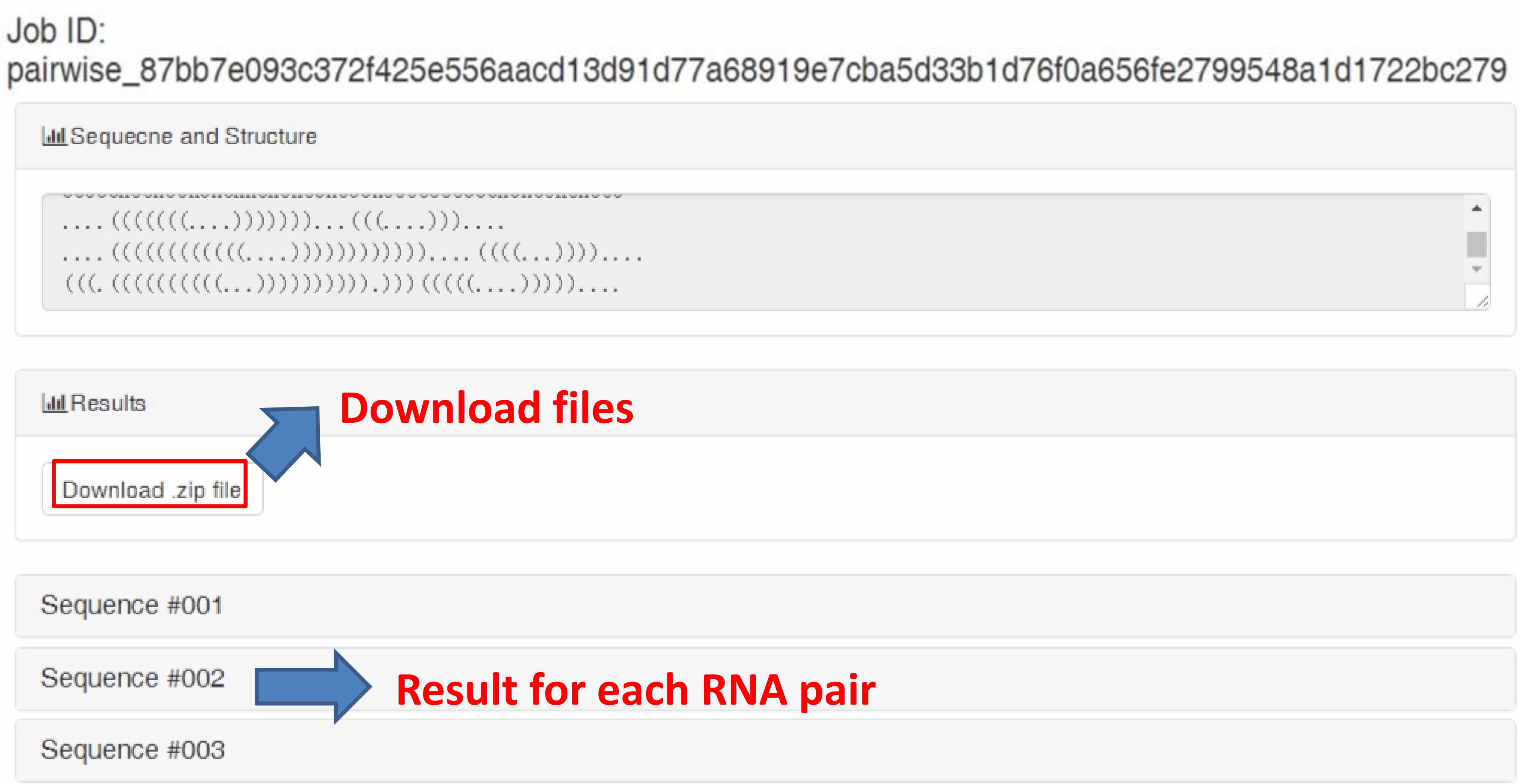

The first pair comparison :ALMV vs ALMV

Sequence #001

The distance score between "ALMV" and "ALMV":
0.000000

Distance score= 0

Structure

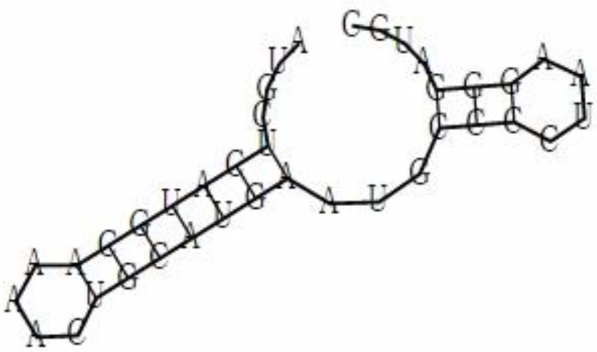

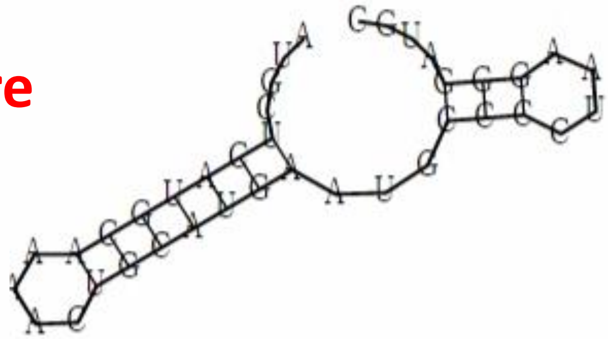

RNA-Structure-1

RNA-Structure-2

TV curve

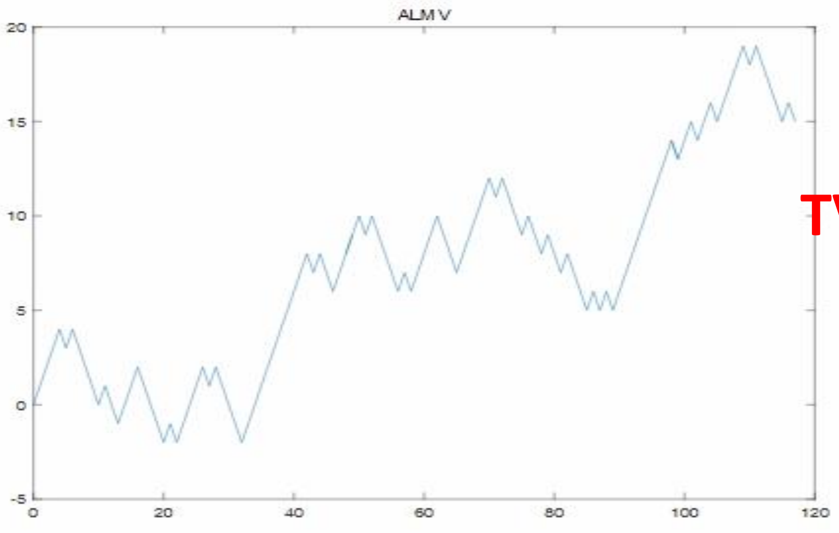

RNA-TVcurve-1

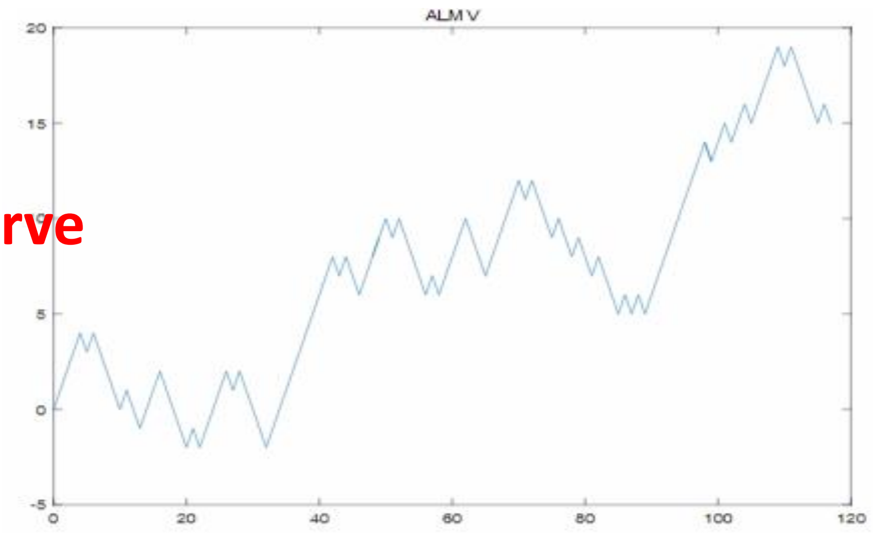

RNA-TVcurve-2

Sequence #003

The third pair comparison :TSV vs LRMV

```
The distance score between "TSV" and "LRMV":
0.835016
```

Distance score=0.835016

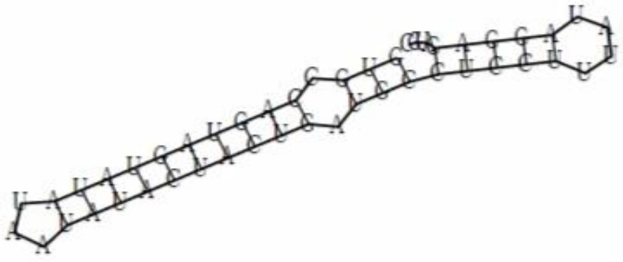

RNA-Structure-1

Structure

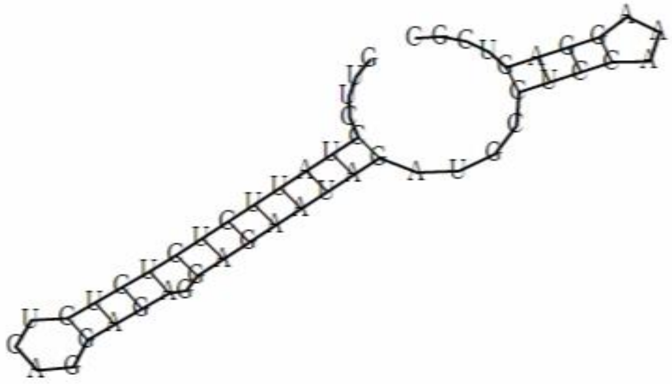

RNA-Structure-2

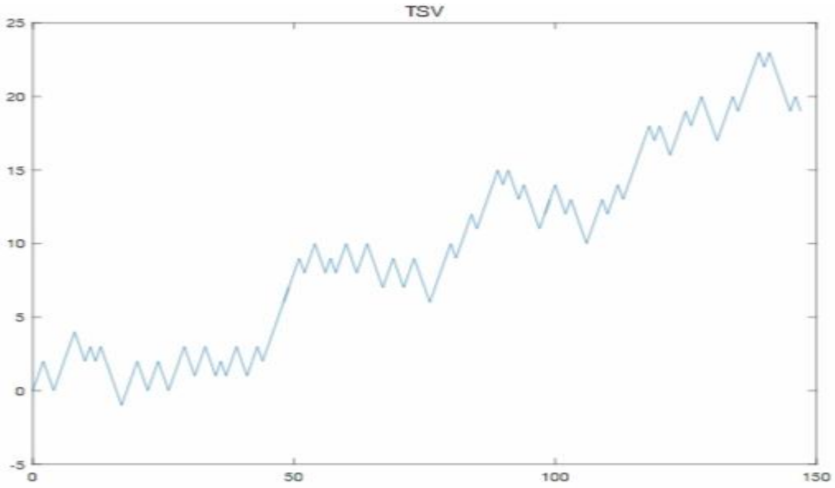

RNA-TVcurve-1

TV curve

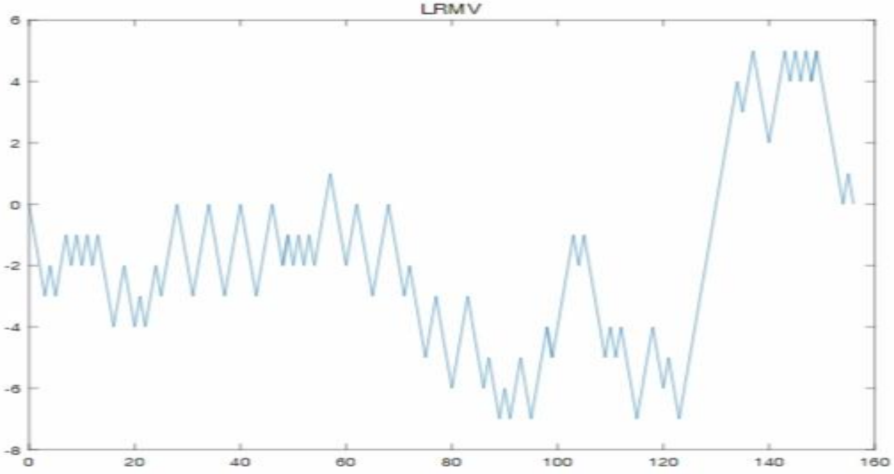

RNA-TVcurve-2
