# Supplementary material for: RNA-TVcurve: a Web server for RNA secondary structure comparison based on a multi-scale similarity of its triple vector curve representation
Source: BMC Bioinformatics. 2017 Jan 21;18:51. doi: 10.1186/s12859-017-1481-7 (PMC5251234; doi:10.1186/s12859-017-1481-7)
Supplement: Additional file 7: Table S1. — The information of families and species of Set 1 of 5S rRNA. Table S2. The information of families and species of Set 2 of RNase P and RNase MRP. (DOC 49 kb) [file 12859_2017_1481_MOESM7_ESM.doc]

**Additional file 1 Table S1.** The information of families and species of Set 1 of 5S rRNA

| Set 1: 5S rRNA | | |
| --- | --- | --- |
| Halobacterium sp. 1 | Euryarchaeota | Archaea |
| Pyrodictium occultum 1 | Crenarchaeot |
| Sulfolobus sp. 1 |
| Dicyema misakiense 1 | Mesozoa | Eukaryotes |
| Actinia equina 1 | Metazoa |
| Chrysaora quinquecirrha 1 |
| Planocera reticulata 1 |
| Basidiobolus magnus 1 | Fungi |
| Christiansenis pallid 1 |

**Additional file 1 Table S2**. The information of families and species of Set 2 of RNase P and RNase MRP

| Synechocystis-PCC6803 RNase P | Cyanobacterial | Bacteria | RNase P |
| --- | --- | --- | --- |
| A.nidulans RNase P |
| Anabaena-PCC7120 RNase P |
| Pseudoanabaena-PCC6903 RNase P |
| R.rubrum RNase P | [Alpha subdivision](http://www.mbio.ncsu.edu/rnasep/alpha-purples.html) |
| A.tumefaciens RNase P |
| B.subtilis RNase P |  |
| T.maritima-g RNase P |  |
| H.cutirubrum RNase P | Euryarchaea | Archaea |
| M.jannaschii RNase P |
| S.acidocaldarius RNase P |  |
| H.sapiens RNase P |  | Eukaryotes |
| P.purpurea-chloroplast RNase P |  |
| | R.americana-mito RNase P | | --- | |  |
| Mus RNase MRP | Muridae | Eukaryotes | RNase MRP |
| Rattus RNase MRP |
| Bos RNase MRP |  |
| Homo RNase MRP |  |
